# Chimeric Rhinoviruses Displaying MPER Epitopes Elicit Anti-HIV Neutralizing Responses

**DOI:** 10.1371/journal.pone.0072205

**Published:** 2013-09-06

**Authors:** Guohua Yi, Mauro Lapelosa, Rachel Bradley, Thomas M. Mariano, Denise Elsasser Dietz, Scott Hughes, Terri Wrin, Chris Petropoulos, Emilio Gallicchio, Ronald M. Levy, Eddy Arnold, Gail Ferstandig Arnold

**Affiliations:** 1 Department of Chemistry and Chemical Biology, Rutgers University, Piscataway, New Jersey, United States of America; 2 Center for Advanced Biotechnology and Medicine, Piscataway, New Jersey, United States of America; 3 Monogram Biosciences, South San Francisco, California, United States of America; Chinese Academy of Medical Sciences, China

## Abstract

**Background:**

The development of an effective AIDS vaccine has been a formidable task, but remains a critical necessity. The well conserved membrane-proximal external region (MPER) of the HIV-1 gp41 glycoprotein is one of the crucial targets for AIDS vaccine development, as it has the necessary attribute of being able to elicit antibodies capable of neutralizing diverse isolates of HIV.

**Methodology/Principle Findings:**

Guided by X-ray crystallography, molecular modeling, combinatorial chemistry, and powerful selection techniques, we designed and produced six combinatorial libraries of chimeric human rhinoviruses (HRV) displaying the MPER epitopes corresponding to mAbs 2F5, 4E10, and/or Z13e1, connected to an immunogenic surface loop of HRV via linkers of varying lengths and sequences. Not all libraries led to viable chimeric viruses with the desired sequences, but the combinatorial approach allowed us to examine large numbers of MPER-displaying chimeras. Among the chimeras were five that elicited antibodies capable of significantly neutralizing HIV-1 pseudoviruses from at least three subtypes, in one case leading to neutralization of 10 pseudoviruses from all six subtypes tested.

**Conclusions:**

Optimization of these chimeras or closely related chimeras could conceivably lead to useful components of an effective AIDS vaccine. While the MPER of HIV may not be immunodominant in natural infection by HIV-1, its presence in a vaccine cocktail could provide critical breadth of protection.

## Introduction

Despite the continued absence of an AIDS vaccine, it is widely agreed that a vaccine must be developed, as it is the most promising strategy for widespread protection against AIDS. The consensus has been that an ideal prophylactic AIDS vaccine will target the earliest events of infection by human immunodeficiency virus (HIV) and activate both the humoral and cellular immune responses [1,2,3] with an emphasis on eliciting broadly neutralizing antibodies, since B-cell responses are likely to confer the greatest long-term protection [4,5,6]. In support of this, passive administration of neutralizing antibodies has provided protection in nonhuman primates challenged with simian HIV (SHIV) [7,8,9,10,11] and has been associated with measurable benefits in controlling viremia in HIV-1-infected humans [12]. Furthermore, immunization studies with various envelope-based constructs have shown immune protection in macaques [13,14,15,16,17] as well as 31.2% efficacy in humans (in the RV144 phase III clinical trial, involving a recombinant viral vector prime followed by an envelope protein boost [18]), offering early glimmerings for hope that improved immunogens might offer greater protection.

The greatest challenge to AIDS vaccine development has been the inability to isolate or engineer safe and broadly neutralizing immunogens that can block infection by the diverse circulating strains of HIV. Efforts to generate the necessary breadth of protection have focused, in large part, on the highly conserved membrane proximal external region (MPER) of gp41. Among the roughly 20 broadly neutralizing antibodies (bnAbs) known to target the envelope glycoproteins gp120 and gp41 [19,20,21], broadly neutralizing antibodies 2F5, 4E10, Z13e1, and more recently, 10E8 target the MPER.

Studies with the 2F5, 4E10, Z13e1, and 10E8 mAbs have helped elucidate the dynamic movement that the MPER normally undergoes in the processes of membrane fusion and viral entry [22,23,24,25,26,27,28,29,30,31]. By bending the MPER at a hinge (2F5, 4E10, and 10E8) or rigidifying the structure of the MPER (Z13e1) [26,27,28,29,31], the MPER-directed antibodies appear to effect neutralization by interfering with the post-CD4 binding steps necessary for formation of the pre-hairpin intermediate [32], most likely via a required initial interaction of their H3 loops with the viral membrane [26,33,34].

Efforts to generate MPER-based immunogens have been particularly challenging, with most efforts yielding little or no neutralization [15,35,36]. More recently, Wang et al. [37] described constructs in which the N- and C-terminal heptad repeats of HIV gp41 were connected via linkers to form six-helix bundles with C-terminal MPER tails. These constructs were able to neutralize primary pseudovirus infection and inhibit syncytium formation and cell-to-cell transmission of virus, but this ability required large doses of purified IgGs. Likewise, Zhou et al. [38] described peptide conjugates encompassing the 2F5 epitope and part of the 4E10 epitope that were able to elicit modest neutralization against Tier 1 HIV isolate SF162.SL. More promising was a report [39] describing the production of chimeric influenza: HIV constructs (in which HIV gp120 was replaced with influenza hemagglutinin (HA), leading to gp41: HA chimeras, some of which were presented on SIVmac239 Gag virus-like particles. One chimera was able to elicit neutralizing antisera in guinea pigs capable of neutralizing three Tier 2 subtype B pseudoviruses.

In this work, we modeled the 2F5 and 4E10 epitopes into a surface loop of human rhinovirus type 14 (HRV14) *in silico* with the goal of minimizing the need for significant conformational reorganization upon binding partner mAbs 2F5 and 4E10. Graphic models were used to design combinatorial libraries in which the epitopes were connected to the HRV loop by short linkers of variable lengths and sequences, optimized to include HIV-like presentations of the epitopes (as has been done previously to generate other immunogenic presentations of HIV epitopes [35,40,41,42]). In the case of the 2F5 epitope presentations, we previously explored novel molecular dynamics simulations approaches that optimized the length, hydrophobicity, and propensity of the epitope for vaccine design [43] aiming to promote the HIV-like type 1 β-turn conformation of this epitope [44]. In the case of the 4E10 epitope, we aimed to promote the known amphipathic α-helical structure of this epitope in its free and antibody-bound conformations. Once generated, the chimeric viruses were subjected to immunoselection protocols using the cognate HIV antibodies to identify the viruses most optimally displaying the inserted epitope. To assess the antigenicity of a guided sampling of the viruses, we used direct and competitive ELISAs (measuring the ability of HIV MPER antibody to bind the MPER-presenting chimeras) as well as a neutralization assay (measuring the ability of HIV MPER antibody to inhibit MPER-presenting chimeric virus infection of cells). After assessing the antigenicity, those deemed to present their HIV epitopes in ways well recognized by 2F5 or 4E10 were chosen to immunize guinea pigs.

One of the chimeras displaying the 2F5 epitope was able to elicit neutralizing responses against pseudoviruses of the AE, F, and, to a lesser extent, D subtypes. Among 10 chimeras designed to display the 4E10 epitope, one elicited neutralizing antibody responses against pseudoviruses of subtypes A, B, C, D, AE, and F (representing all of the subtypes tested) while four others elicited significantly neutralizing antibodies against pseudoviruses of the AE, F, and, to a lesser extent, A, B, or D subytpes. While the neutralization titers obtained were modest, this work confirms that HRV chimeras displaying MPER sequences can induce broad anti-HIV-1 neutralizing responses, and could possibly be further optimized to elicit more potent immune responses.

## Materials and Methods

### Cells, viruses, media, bacteria and plasmids

H1-HeLa cells [45] were used to produce, propagate, titer, and immunoselect wild-type HRV14 as well as the recombinant HRV14: HIV-1 gp41 2F5 and 4E10 chimeric viruses. *M* *medium* [46] with 1-10% fetal bovine serum was used for propagation of the viruses; PA medium [46] with 1-10% fetal bovine serum was used for isolation and titering of the viruses; Dulbecco’s modified Eagle medium (DMEM; Gibco, BRL, Carlsbad, CA) was used for HeLa cell transfection; and AH medium [46] was used for large-scale propagations of the viruses. The bacterial strain used for transformation of the engineered plasmids was *E. coli* DH10B ElectroMax (Gibco, BRL, Carlsbad, CA). The pST-LIC plasmid was used for all of the genetic engineering. This plasmid was derived from modification of the previously used HRV-encoding p3IIST plasmid [47], whereby the sequences flanking the missing VP2 puff of the NIm-II region of HRV14 [48] were modified to have extended sticky ends to maximize the efficiency of ligation with complementary HIV-encoding DNA oligonucleotides [42].

### Antibodies and peptides

The broadly neutralizing human mAbs, 2F5 (PolyMun, Inc., Vienna, Austria), which binds the gp41 MPER epitope E^662^LDKWA^667^ [49], 4E10 (PolyMun, Inc., Vienna, Austria), which binds the gp41 MPER epitope, N^671^WFDITNW^678^, and Z13e1 (Michael Zwick, The Scripps Research Institute, La Jolla, CA), which binds to the gp41 MPER epitope, N^671^WFDIT^676^, were used for immunoselections and, in the case of 2F5 and 4E10, antigenicity assays. Antibodies used for ELISAs included anti-HRV14 murine mAbs 1 and 17 [which bind to the neutralizing immunogenic site IA (NIm-IA) [50] and were provided by Dr. Roland Rueckert, University of Wisconsin, Madison, WI], horseradish peroxidase (HRP)-conjugated goat-anti-mouse IgG (Cappel ICN, Irvine, CA), and Fc-specific sheep anti-mouse IgG (Sigma, St. Louis, MO). Ac-EQELLELDKWASLW-NH_2_ peptide (synthesized by NeoMPS, Inc., San Diego, CA) and the K7-D11 lactam-bridged Ac-NWFDITK _7_WLWD _11_KKK-NH_2_ (NK-15) peptide, (purchased from Chempep, Inc., Wellington, FL but originally synthesized and characterized by Michel Sun in the laboratory of Dr. John W. Taylor, Department of Chemistry and Chemical Biology at Rutgers University) were employed for competition in ELISAs and for elution of the chimeric viruses in competitive immunoselections. Three keyhole limpet hemocyanin (KLH)-conjugated peptides were used for immunization of guinea pigs: KLH-EQELLELDKWASSLW-NH_2_ (KLH-P-E; NeoMPS, Inc., San Diego, CA), KLH-EQELLALDKWASSLW-NH_2_ (KLH-P-A; NeoMPS, Inc., San Diego, CA), and KLH-C‑NWFDITK _7_WLWD _11_KKK-NH_2_ (KLH-NK-15; Chempep, Inc., Wellington, FL).

### Production of the chimeric HRV14: ELDKWA (2F5) and HRV14: NWFDITKWLW (4E10) libraries

**Figure 1:**
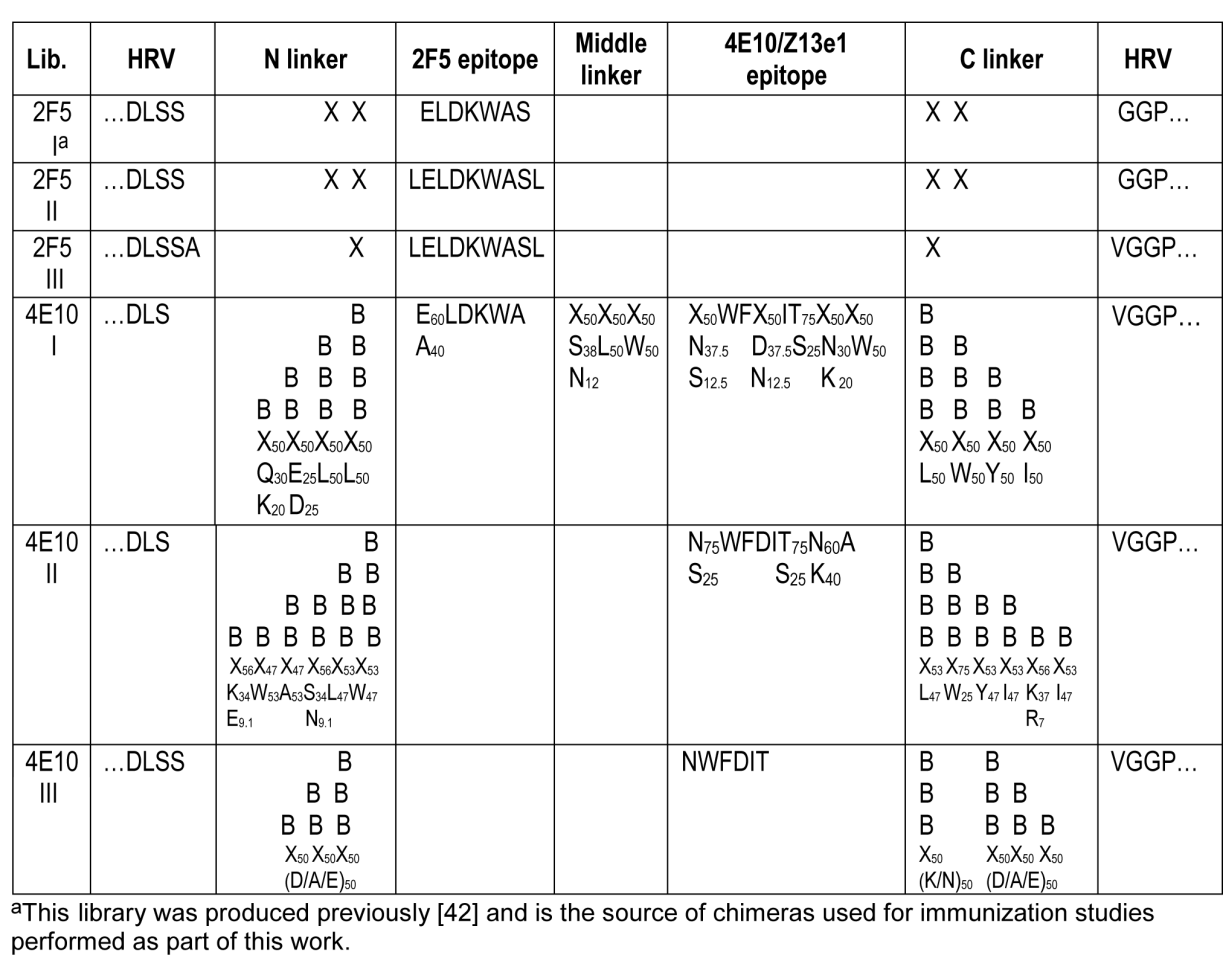
Design of 2F5- and 4E10 libraries produced in effort to obtain useful HIV-1 MPER-displaying immunogens. X = any of the 20 amino acids. B’s indicate biasing of residues (to represent mixtures of the HIV-1 residues found at the given site (in proportion to their representation in the Los Alamos Database) with randomized residues (X) to allow for other solutions that would be compatible with virus viability and optimal presentation of the epitope. One exception to using the HIV residues in the mixture is the use of an equimolar mixture of D/A/E in Library III; this was chosen in the 4E10 Lib III to promote α-helix formation. Subscripts correspond to relative percentages encoded (in the case of the HIV residue, reflecting the values among HIV-1 isolates at the time of the library designs).

The libraries produced for this work are shown in Figure 1. For each combinatorial library, a complex set of complementary recombinant DNA oligonucleotides was synthesized (all by IDT, Piscataway, NJ). The oligomers were designed to overlap in the epitope-encoding region using codons preferred by the HRV14 RNA polymerase. Sets of complementary epitope-encoding DNA oligonucleotides were hybridized together and extended to fill in the variable-sequence overhangs on both ends [using each of the dNTPs and Klenow DNA polymerase I (New England Biolabs, Beverly, MA)]. The resultant double-stranded cassettes were then treated with bacteriophage T4 DNA polymerase (Sigma, St. Louis, MO) and dGTP to generate the complementary 11-15 base sticky ends due to the 3’–5’ exonuclease activity of the T4 DNA polymerase in the presence of dGTP. The HRV14-encoding pST-LIC plasmid was uniquely digested in the region for insertion with BseRI and then treated with T4 DNA polymerase and dCTP to generate long complementary sticky ends. A ligation-independent cloning method was used in which the sticky-ended ELDKWA-encoding oligonucleotides were hybridized to the sticky-ended vector in a 10:1 ratio at 22°C for 1 h. The recombinant plasmids were amplified by their electroporation into DH10B *E. coli* cells using a Gene Pulser system (Bio-Rad laboratories, Hercules, CA) at 1.8 kV, 200 ohms, and 25 µF in 0.1 cm pre-chilled cuvettes). Transformed cells were grown in bulk liquid cultures at 30-37°C to late log phase and then harvested using HiSpeed Mini- or Midi-Prep Spin Kits (Qiagen, Valencia, CA). After purifying and linearizing the pools of recombinant plasmids with MluI, the plasmids were transcribed *in vitro* using Ambion MegaScript kits (Austin, TX). Full-length RNA transcripts were transfected into H1-HeLa cells by electroporation (using 10 µg/10 µL of the infectious RNAs added to 1 X 10^7^ H1-HeLa cells in 400 µL of D-MEM in 0.4 cm Gibco-BRL electroporation cuvettes [using a Gene Pulser system (Bio-Rad laboratories, Hercules, CA) at 250 V, and 950 µF]. The resultant chimeric viruses were grown by plating the transfected cells with an equal number of unpulsed cells for virus propagation, incubating at 34.5°C for 3-4 days, and harvesting when the cytopathic effect (CPE) reached approximately 90% as previously described [46].

### Partial purification of virus libraries and virus isolates

A protocol developed by Zhang et al. [51] was followed for the partial purification (“mini purification”) of the combinatorial virus libraries as well as for individual viruses. Briefly, after three cycles of freezing and thawing, the concentrated harvested suspensions of infected cells were centrifuged (to remove cell wall debris), after which the clarified lysates were subjected to DNase I treatment followed by ultracentrifugation (with a 30% sucrose cushion) at 42,000 rpm for 2.5 h at 15°C in a Beckman 45 Ti rotor. The pellets, which contained the chimeric viruses as well as some cell-derived ribosomes, were resuspended in 20 mM Tris-HCl, pH 7.4 (sometimes with 100-150 mM NaCl added to improve solubilization).

### Immunoselection of viruses

Immunoselection was performed as previously described [35,47] with some modifications. Ninety-six-well Immunosorp plates (Nunc, Rochester, NY) were coated with mAb 2F5 (using a 10 nM solution) or 4E10 (using a 0.84, 6.7, or 13.3 nM solution) to capture chimeric rhinovirus pools with conformations well recognized by the mAb, essentially as described previously [41]. After overnight incubation at 4°C, plates were blocked with 3% gelatin in phosphate-buffered saline (PBS) or 4% nonfat dry milk in PBS for 1 h at 37°C.

#### Without peptide competition

Plates were washed six times with PBS/0.05%-0.1% Tween 20 (PBS-T) and 3 X 10^5^ plaque-forming units (PFU)/well of chimeric viruses were added and incubated for 2 h at 34.5°C and 2.5% CO_2_. Plates were washed five times with PBS-T and three times with PBS only. Antibody-bound virus was eluted either with H1-HeLa cells alone or with peptide followed by cells. When peptide was used (as was the case with some of the 4E10 sub-libraries), 750 to 3000 µM of NK-15 was used in the first rounds and 750-6000 µM in the 2nd round of immunoselection (incubated for 2 h at 37°C, then washed six times with 0.1% PBS-T). In the absence of peptide-induced release of chimeras and in the cases following peptide-induced release, 2 X 10^4^ H1-HeLa cells were seeded into the wells using medium M with 10% FBS per well. Plates were then incubated at 34.5°C, 2.5% CO_2_ for up to 72 h (until cells reached 80-<100% CPE). The harvested cells were lysed by three cycles of freezing and thawing and then centrifuged to produce clarified virus lysates.

### With peptide competition

Competitive immunoselection was performed like immunoselection without peptide competition, with one modification. Prior to the addition of virus to the antibody-coated plates, virus was preincubated for 1 h at room temperature with peptide. In the case of the 2F5 libraries, 0, 2, 4, 8, and 16 nM of competitive peptide Ac-EQELLELDKWASSLW-NH_2_ were used; in the case of the 4E10 libraries, 0-320 nM of competitive peptide NK-15 was used in the first round and 0-640 nM in the 2nd round of immunoselection.

### Enzyme-linked immunosorbent assays (ELISAs) of library pools and chimeric viruses

#### Direct ELISAs

Direct enzyme-linked immunosorbent assays (ELISAs) were performed in triplicate on individual viruses and virus pools to assess virus binding to 4E10 antibody. In brief, 96-well Maxisorp plates (Nunc, Denmark) were coated with 0.2 µg/well of 4E10 monoclonal antibody in 50 mM sodium borate, pH 8.5 at 4°C overnight, and then blocked with 4% non-fat dry milk in phosphate-buffered solution (PBS) for 1 h at 37°C. After washing the plates six times with PBS/0.1% Tween 20 (PBS-T), serially diluted chimeric viruses or virus pools were added into the wells and incubated for 2 h at 37°C. Plates were washed and 0.4 to 1.3 µg/ml of anti-HRV14 mAb 1 was added, incubated for 1 h at 37°C, washed six times, and treated with HRP-conjugated sheep anti-mouse IgG. Then, peroxidase substrate (0.3 mg/ml tetramethylbenzidine dissolved in 10% dimethyl sulfoxide in 0.18 M sodium citrate, pH 3.95) was added. The reaction was catalyzed by the addition of H_2_O_2_ to 0.009%, allowed to develop color, and then stopped by the addition of an equal volume of 1 M H_2_SO_4_. Titers are expressed as reciprocals of virus stock dilutions (relative to an initial concentration of 1 X 10^8^ PFU/ml) at which an optical density at 450 nm (OD_450_) of 0.5 was achieved. All data are reported as the average of three measurements, with the errors shown as the standard errors of the mean.

#### Competitive ELISAs

Competitive ELISAs were performed in triplicate using immobilized mAb 2F5 or 4E10 according to Arnold et al. [35] with the exception that 3 X 10^5^ PFU virus were mixed with either the 14-mer ELDKWA-containing peptide or the NK-15 peptide in serial dilutions before being added to immobilized 2F5 or 4E10 for 2 h at 37°C. The anti-HRV14 mAb 17 was added, and the samples were incubated and then washed prior to treatment with HRP-conjugated goat anti-mouse IgG. The samples were further incubated, washed, and treated with peroxidase substrate. The color reactions were stopped and read as above. All data are reported as the average of three measurements, with the errors shown as the standard errors of the mean. The competing peptide was used to promote more stringent binding of mAb to those chimeras displaying the epitope in conformations better resembling those of the native epitope than the peptide.

### Chimeric virus MTT neutralization assay

Chimeric virus MTT neutralization assays were performed as previously described [35,47]. Briefly, 50 µl of 2 X 10^5^ PFU/ml of chimeric viruses was incubated in *M* *medium* for 1 h at 34.5°C, 2.5% CO_2_ with 50 µl of sequential two-fold dilutions of mAb (starting at a 4 µg/ml concentration) in 96-well tissue-culture microtiter plates (Nunc, Rochester, NY. 50 µl of H1-HeLa cells was added at a concentration of 2 X 10^5^ cells/ml. The plates were incubated at 34.5°C, 2.5% CO_2_ for up to 48 h. 15 µl of a 5 mg/ml solution of 3-(4,5 dimethylthiazol)-2-yl-2,5-diphenyltetrazolium bromide (MTT; Sigma-Aldrich, St. Louis, MO) in PBS was added to the wells. After a 1.5 h incubation at 34.5°C, 2.5% CO_2_, the reaction was stopped by adding 150 µl of 20% sodium dodecyl sulfate in 50% N,N-dimethylformamide. Cell survival was defined as the percentage of the OD_570_ observed compared to that of the cell control wells, which displayed 100% viability. The antibody 50% inhibitory concentration (IC_50_) values correspond to the mAb concentration necessary to neutralize chimeric virus infection by 50%. All data are reported as the average of three measurements, with the errors shown as the standard errors of the mean.

### Plaque isolation and sequencing of chimeric viruses

Individual virus plaques were subjected to two rounds of plaque purification according to Smith et al. [41]. The chimeric viruses were then propagated in 60 mm dishes. Viral RNAs were isolated using QIAamp Viral RNA Purification Kits (Qiagen, Valencia, CA) and RT-PCR was performed using QIAquick Gel Extraction Kits (Qiagen, Valencia, CA) on electrophoresed PCR products. The DNA fragments were then sequenced (IDT, Piscataway, NJ).

### Transmission Electron Microscopy

The mini-purified chimeric viruses were diluted 100-fold with 100 mM Tris-HCl, pH 7.4. Formvar/Carbon grids were each treated with a drop of virus solution and then stained with 1% phosphotungstic acid for 5 minutes. The grids were washed 5 times with distilled water, air dried for 5 minutes, and then observed using a JEOL 1200EX scanning/transmission electron microscope.

### Immunization of guinea pigs

Young male Dunkin Hartley guinea pigs were immunized subcutaneously with each chosen chimeric virus (using three guinea pigs/chimera). The immunizations were carried out as follows: weeks 0 and 4: 40 µg (approximately 1 X 10^9^ PFU) of mini-purified chimeric virus in either 10 mM Tris-HCl or -HAc, pH 7.4; for weeks 9 and 13: 40 µg of mini-purified chimeric virus in 10 mM Tris-HCl or -HAc, pH 7.4 boosted with corresponding keyhole limpet hemocyanin (KLH)-conjugated peptide. (For the ELDKWA chimeras (B1 and C1), the peptide boosts consisted of 80 µg each of KLH-EQELLELDKWASSLW-NH_2_ (KLH-P-E) and KLH-EQELLALDKWASSLW-NH_2_ (KLH-P-A), mixed with an equal volume of complete Freund’s adjuvant (CFA) for week 9 and an equal volume of incomplete Freund’s adjuvant (IFA) for week 13. For the NWFDITKWLW chimeras, the peptide boosts consisted of 120 µg KLH-conjugated 16-mer K8-D12 lactam-bridged peptide KLH-C-NWFDITKWLWDKKKK-NH_2_ (KLH-NK-15) mixed with an equal volume of CFA for week 9 and an equal volume of IFA for week 13. In all of the physiological solutions tested, the NWFDITKWLW peptide was predominantly dimeric. Sera were collected by femoral bleeds or heart sticks at weeks -1, 7, 12, and 16.

### Ethics statement

This study was carried out in strict accordance with the recommendations in the Guide for the Care and Use of Laboratory Animals of the National Institutes of Health. The protocol was approved by the Office of Laboratory Animal Welfare (OLAW) and submitted in compliance with the Public Health Service (PHS) Policy on Humane Care and Use of Laboratory Animals (Identification Number: A3669-01). All blood collections were performed with ketaset and xylazine, and technicians were trained to minimize suffering by the animals.

### HIV-1 pseudovirus neutralization assay

We assessed the ability of the sera from chimera-immunized guinea pigs to neutralize recombinant HIV-1 pseudoviruses of diverse subtypes and co-receptor usages using a single-round replication assay [52] developed at Monogram Biosciences, Inc. (South San Francisco, CA). The pseudoviruses were preincubated with various dilutions of heat-inactivated guinea pig sera for 18 hours and then added to U87 cells expressing the CD4 receptor and the CCR5 and CXCR4 co-receptors. Neutralizing titers were calculated after one round of replication as the reciprocal of the guinea pig serum dilution yielding 40% inhibition of the reporter luciferase activity (compared with pooled normal guinea pig serum values).

## Results

### Construction and production of three combinatorial ELDKWA/2F5 libraries

#### 2F5 Library I

In an effort to produce live-virus immunogens capable of eliciting effective and cross-reactive immune responses against HIV-1, we have made a number of combinatorial libraries of chimeric viruses that display the conserved 2F5 epitope, E^662^LDKWA^667^ [49,53], of the HIV-1 membrane-proximal external region (MPER; [35,42]; and this work). This region of the MPER appears to be flexible, normally assuming an extended structure in the N-terminal portion and a helical structure in the C-terminal portion, becoming more extended with β-turn conformations in the presence of 2F5 [31,43,44,54,55,56,57]. The extension of the ELDKWA residues appears to be initiated by the binding of the CDR H3 loop of 2F5 to the adjacent 4E10 epitope in the vicinity of residues 669-672 [27,29,31,58], lifting L669 and W670 out of the membrane [29].

In previous work [42], we produced a subset of a combinatorial library for which the design was enhanced by molecular modeling, using simulations that illustrated the importance of optimizing such key features as the length, hydrophobicity, and conformational propensity of the ELDKWA sequence that was to be inserted in the VP2 puff [59], the major loop of neutralizing immunogenic site II of HRV14 [50,60]. Given that numerous ELDKWA peptides bound to 2F5 are characterized by having a predominant type 1 β turn [44], our goal was to produce a type 1 β-turn conformation upon inserting the ELKDWA epitope onto the surface of HRV14. We assumed that predisposing the ELDKWA epitope to bind to 2F5 would translate to an increased ability of the chimeras to bind 2F5 and, therefore, elicit 2F5-like antibodies upon immunization of animals.

We performed this work with several ELDKWA chimeras generated previously (namely B1-16, B2-16, C1-8, D1-4, and F1-0 [42]) as well as with additional chimeras that were generated anew from the same library (2F5 Library I, Figure 1). We also designed, generated (when possible), and selected chimeras from a number of additional libraries encoding the 2F5 epitope (2F5 Libraries II and III, Figure 1). In each case, the epitope was inserted in such a way as to replace a small segment of the VP2 puff, the site engineered most successfully in our previous work [35,40,41].

**Figure 2:**
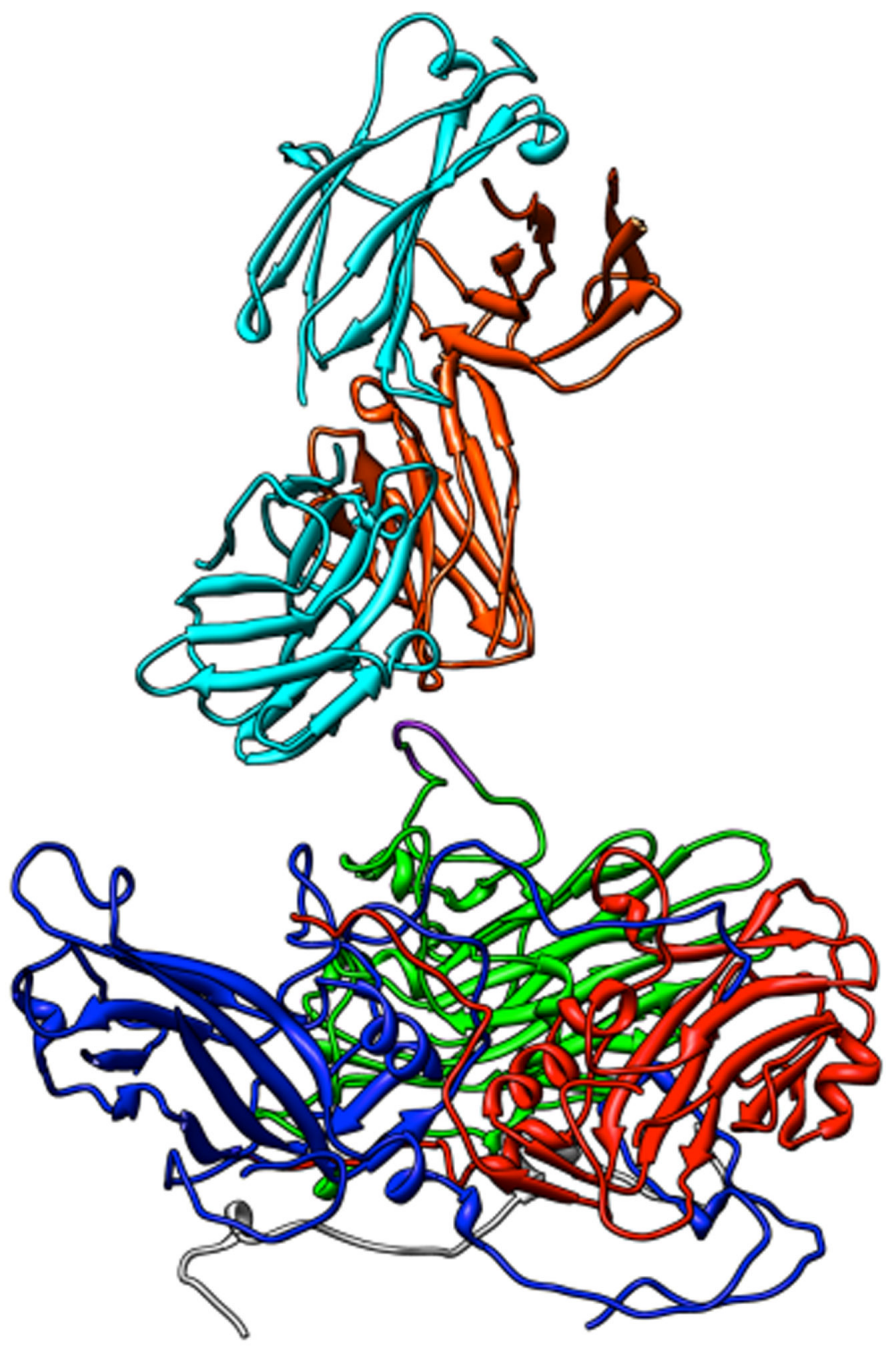
The ELDKWA epitope of chimeric virus B1-16 (shown in purple) docked with the surface of the 2F5 paratope. The protomeric unit of HRV, 60 of which come together to make the viral capsid, is in the bottom of the image and one of the Fv domains of the 2F5 antibody is in the top. The viral proteins are colored blue for VP1, green for VP2, and red for VP3, and the heavy and light chains of 2F5 are respectively in orange and light blue.

Using advanced molecular dynamics methods to sample and cluster epitope conformations for optimization of their conformational propensity and exposure, we arrived at a predicted structure for B1-16 [42], illustrated in Figure 2. This chimera was generated from 2F5 Library I, for which DNA oligonucleotides were synthesized that encoded the ELDKWAS sequence flanked on either side by two randomized amino acids (encoding any of the 20 commonly occurring amino acids), enhancing the chances of identifying chimeras with favorable growth characteristics and well presented ELDKWAS inserts. The DNA oligonucleotides were hybridized, extended in the region of the unique, randomized sequences, and ligated into a plasmid, pST-LIC, that encodes HRV14 (except for the region corresponding to the VP2 puff, to ensure that only recombinant plasmids would be capable of producing infectious RNAs). The recombinant plasmids were amplified in bacteria and then purified for use as templates for *in vitro* transcription reactions.

This purified ELDKWAS-encoding chimeric HRV plasmid library was subjected to *in vitro* transcription to produce infectious RNAs. After transfection of the RNA library into H1-HeLa cells, a pool of chimeric viruses was produced, containing approximately 6 X 10^3^ independent viruses (measured as a function of the number of plaques obtained by transfection). The chimeric virus pool was propagated and purified at this stage so that viruses selected for further study might be more easily purified at later stages.

#### 2F5 Library II

A second library was designed to be like the 2F5 Library I with the addition of the leucine residues that flank the ELDKWAS epitope among most HIV-1 isolates (2F5 Library II, Figure 1). The inclusion of the leucines was based on the observation that the Ac-LELDKWASL-NH_2_ peptide was seen to bind 2F5 with nearly three orders of magnitude greater affinity than the Ac-ELDKWAS-NH_2_ peptide [61]. Library II was produced using the same methods as those used to produce Library I. Oddly, only a single chimeric virus sequence was found in this library among five sequenced at the plasmid level.

#### 2F5 Library III

A third library was designed with the extended LELDKWASL sequence, this time removing one of the randomized flanking residues on each side (2F5 Library III, Figure 1). Library III was produced using the same methods as those used to produce Libraries I and II. Production of this library also led to an unexpected result: while the plasmids generated were found to be normal, no viruses were obtained from the plasmids using conditions that normally would have yielded hundreds of viable viruses.

### Immunoselection of Library I

**Figure 3:**
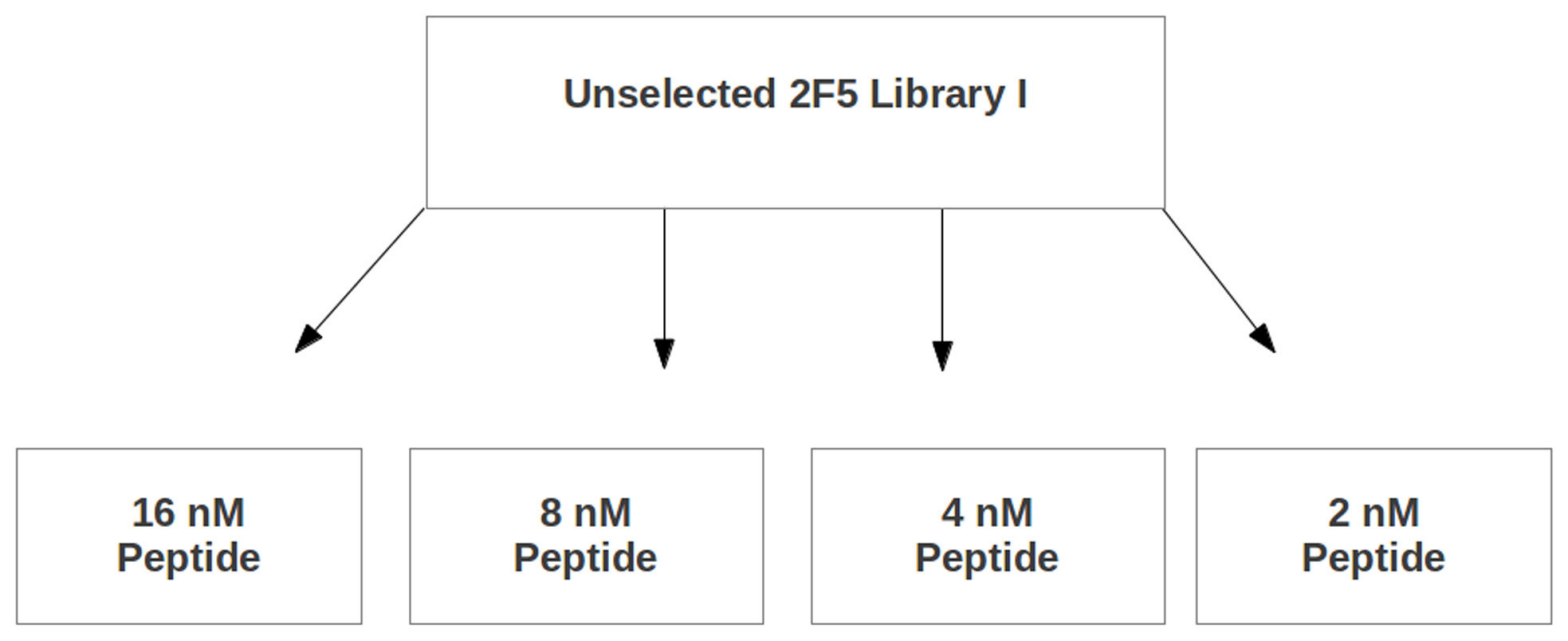
Docking of HRV14 (shown in yellow) with a crystallographic structure (1TZG from the Protein Data Bank) of a complex of the 4E10 Fab (orange) complexed with the cognate Ac-KGWNWFDITNWGK-NH_2_ peptide (blue ribbon and gold stick-and-balls model). Docking provides ideas for how to connect the 4E10 epitope to the surface of HRV.

In order to select for viruses that would ideally present their ELDWAS sequences in ways resembling those of HIV-1, viruses from Library I were immunoselected in one round on the basis of their ability to bind to immobilized mAb 2F5 in the presence of 0-32 pmol of competing peptide (Ac-EQELLELDKWASLW-NH_2_; Figure 3). The chimeric viruses thus selected grew at rates comparable to that of wild-type HRV14, indicating that the presence of the inserts was not deleterious to their growth. We observed a non-random preponderance of P and G residues among the flanking residues on both sides of the insert, with both P and G known for promoting β-turns (and with Ps also known for disrupting β sheets; Table 1). This might be related to structural features of the engineered loop that affect the stability or acceptable folding of the chimeric viruses that are compatible with the ability to bind 2F5 [42]. As with their unselected counterparts, the sequenced viruses were all seen to have intact ELDKWAS epitopes.

**Table 1:** Sequences of the most antigenic chimeras immunoselected and characterized from 2F5 Libraries I and II.

**Chimeric viruses**	**HRV**	**N linker**	**Core epitope**	**C linker**	**HRV**
**ELDKWAS Library I Chimeras**					
**B1-16**	… DLSS	HG	ELDKWAS	PN	GGP…
**B2-16**	… DLSS	GK	ELDKWAS	QP	GGP…
**C1-8**	… DLSS	PG	ELDKWAS	IP	GGP…
**D1-4**	… DLSS	SP	ELDKWAS	LP	GGP…
**F1-0**	… DLSS	PP	ELDKWAS	SP	GGP…
**Lib 1-1**	… DLSS	PG	ELDKWAS	SR	GGP…
**Lib 1-2**	… DLSS	GV	ELDKWAS	AP	GGP…
**Lib 1-3**	… DLSS	PG	ELDKWAS	PN	GGP…
**ELDKWAS Library II Chimera**					
**Lib 2-1**	… DLSS	SS	LELDKWASL	HP	GGP…

2F5 Library III did not produce live viruses.

### Antigenic characteristics of isolated chimeric viruses

A molecular dynamics study was directed toward maximizing the conformational propensity for the β-turn conformation of the epitope inserted onto the chimeric HRV. Affinity measurements obtained from direct and competitive enzyme-linked immunosorbent assays (ELISAs; using 2-16 pmol of Ac-EQELLELDKWASLW-NH_2_ peptide/well), confirmed our predictions that chimeras immunoselected in the presence of the greatest concentration of peptide were able to best bind immobilized mAb 2F5 ( [42] and unpublished data). Consistent with this observation were chimera-antibody binding data from quantitative fluorescence-quenching experiments [42]. Two chimeras, B1-16 and C1-8, showed the greatest affinity to the immobilized 2F5.

### Analysis of the chimeric virus sequences

Viral RNAs were reverse transcribed and sequenced to confirm the presence of ELDKWAS sequences. Despite the fact that only one chimeric virus was obtained from Library II and no chimeras were produced from the plasmids generated from Library III, all chimera sequences that were obtained from Library I were found to be appropriate and without apparent mutations (Table 1).

### Immunization of guinea pigs and characterization of immunogenicity of chimeric viruses

Given their favorable antigenic profiles [42], chimeras B1-16 and C1-8 from Library I were chosen for immunization studies to see if they could elicit 2F5-like antibodies in guinea pigs. We immunized three guinea pigs with each chimera using a prime-boost strategy. Immunizations consisted of subcutaneous injections of 40 µg of mini-purified virus at weeks 0 and 4 followed by immunizations at weeks 9 and 13 with 40 µg of mini-purified chimeric virus and 80 µg of a keyhole limpet hemocyanin (KLH)-conjugated C-EQELLELDKWASLW-NH_2_ peptide. Sera were collected at week 16, after which their neutralizing abilities were tested against 12 HIV-1 pseudoviruses that contained envelope sequences from diverse subtypes (A, B, C, D, AE, and F) and with varying co-receptor usages (X4 and R5). It should be noted that the chimeric HRVs do not replicate in guinea pigs, making them only a rough screen for the types of immune responses that might be possible upon immunizing with live viruses in people.

Table 2 shows the overall neutralization activity of six serum samples elicited from two ELDKWA-displaying chimeric viruses. Positive controls consisted of serum from an HIV-1-positive patient, Z23, as well as purified mAb IgGs 2F5 and 4E10; negative controls consisted of pooled serum from six guinea pigs immunized with a non-engineered HRV14 and the non-HIV-1 pseudovirus, amphotropic murine leukemia virus (αMLV). The neutralization titers are expressed as reciprocal titers at which 40% inhibition of neutralization was observed (compared with pooled normal guinea pig serum values). While some neutralization elicited 50% inhibition, a 40% cut-off allowed us to identify more chimeric viruses that could be worthy of optimization for future immunogen development.

**Table 2:** Neutralization of HIV-1 pseudoviruses from antisera collected from guinea pigs immunized with chimeric viruses (and boosted with MPER-encoding peptides).

	**Pseudovirus (subtype, co-receptor usage)**												
**Chimera: IgG**	**92RW009 (A, X4)**	**92UG037 (A, R5)**	**MN (B, X4)**	**NL43 (B, X4)**	**QZ4589 (B, R5)**	**JRCSF (B, R5)**	**98CN006 (C, R5)**	**98IN022 (C, R5)**	**92UG038 (D, X4)**	**92UG005 (D, R5)**	**92TH024 (AE, R5)**	**93BR029 (F, R5)**	**Control**: α **-MLV**
**2F5: ELDKWA**													
**B1-16: GFA-292**	<20	<20	<20	<20	<20	<20	<20	<20	<20	<20	**70**	**30**	<20
**B1-16: GFA-293**	<20	<20	<20	<20	<20	<20	<20	<20	<20	**80**	**80**	**30**	<20
**B1-16: GFA-294**	<20	<20	<20	<20	<20	<20	<20	<20	<20	20	**80**	50	<20
**C1-8: GFA-289**	<20	<20	<20	<20	<20	<20	<20	<20	<20	<20	<20	<20	<20
**C1-8: GFA-290**	<20	<20	<20	<20	<20	<20	<20	<20	<20	<20	<20	<20	<20
**C1-8: GFA-291**	<20	<20	<20	<20	<20	<20	<20	<20	<20	<20	<20	<20	<20
**4E10: NWFDIT(K/N)**													
**01A3: GFA-286**	<20	<20	<20	<20	<20	<20	<20	<20	<20	<20	20	<20	<20
**01A3: GFA-287**	<20	<20	<20	<20	<20	<20	<20	<20	<20	<20	<20	<20	<20
**01A3: GFA-288**	<20	<20	<20	<20	<20	<20	<20	<20	<20	<20	<20	<20	<20
**02A1: GFA-280**	<20	<20	<20	<20	<20	<20	<20	<20	<20	**25**	**45**	**35**	<20
**02A1: GFA-281**	<20	<20	<20	<20	<20	<20	<20	<20	<20	<20	<20	20	<20
**02A1: GFA-282**	<20	<20	<20	<20	<20	<20	<20	<20	<20	<20	**35**	**35**	<20
**02B2: GFA-283**	<20	<20	<20	<20	20	<20	<20	<20	<20	<20	**35**	<20	<20
**02B2: GFA-284**	<20	<20	<20	<20	<20	<20	<20	<20	<20	<20	<20	<20	<20
**02B2: GFA-285**	<20	<20	<20	<20	**25**	<20	<20	<20	<20	**35**	**60**	**40**	<20
**11A2: GFA-271**	<20	<20	<20	<20	<20	<20	<20	<20	<20	<20	<20	<20	<20
**11A2: GFA-272**	<20	<20	<20	<20	<20	<20	<20	<20	<20	<20	<20	<20	<20
**11A2: GFA-273**	<20	<20	<20	<20	**25**	<20	<20	<20	<20	<20	<20	**40**	<20
**13A3: GFA-274**	<20	<20	<20	<20	<20	<20	<20	<20	<20	<20	<20	<20	<20
**13A3: GFA-275**	<20	<20	<20	<20	<20	<20	<20	<20	<20	<20	<20	<20	<20
**13A3: GFA-276**	<20	<20	<20	**875**	<20	<20	<20	<20	<20	<20	<20	<20	<20
**12B1: GFA-277**	<20	<20	<20	<20	<20	<20	<20	<20	<20	<20	**30**	<20	<20
**12B1: GFA-278**	**45**	**60**	<20	<20	**30**	**50**	**35**	**40**	**40**	**45**	**80**	**50**	20
**12B1: GFA-279**	<20	**30**	<20	<20	<20	**70**	<20	20	<20	20	**90**	**70**	**25**
**13C2: GFA-268**	<20	<20	<20	<20	<20	<20	<20	<20	<20	<20	<20	<20	<20
**13C2: GFA-269**	<20	<20	<20	<20	<20	<20	<20	<20	<20	<20	<20	<20	<20
**13C2: GFA-270**	<20	<20	<20	<20	<20	<20	<20	<20	<20	<20	<20	<20	<20
**21B1: GFA-262**	<20	<20	<20	<20	<20	<20	<20	<20	<20	<20	<20	20	<20
**21B1: GFA-263**	<20	<20	<20	<20	<20	<20	<20	<20	<20	<20	<20	<20	<20
**21B1: GFA-264**	<20	<20	<20	<20	<20	<20	<20	<20	<20	<20	<20	20	<20
**21D1: GFA-265**	<20	**30**	<20	**30**	<20	**35**	<20	20	<20	**35**	**70**	**40**	**40**
**21D1: GFA-266**	<20	<20	<20	<20	<20	<20	<20	<20	<20	<20	<20	<20	<20
**21D1: GFA-267**	<20	<20	<20	<20	<20	<20	<20	<20	<20	<20	<20	<20	<20
**22C1: GFA-259**	<20	<20	<20	<20	<20	<20	<20	<20	<20	<20	<20	**25**	<20
**22C1: GFA-260**	<20	<20	<20	<20	<20	<20	<20	<20	<20	<20	**25**	**70**	<20
**22C1: GFA-261**	<20	<20	<20	20	20	**45**	<20	20	<20	**45**	**50**	**85**	<20
**Controls**													
**HRV14**	**<20**	**<20**	**<20**	**<20**	**<20**	**<20**	**<20**	**<20**	**<20**	**<20**	**<20**	**<20**	**<20**
**2F5 (µg/ml)**	**3.6**	**0.2**	**0.006**	**0.016**	**0.04**	**0.28**	**>100**	**>100**	**1.0**	**1.2**	**0.04**	**0.17**	**>100**
**4E10 (µg/ml)**	**5.5**	**0.66**	**0.02**	**0.19**	**0.12**	**1.3**	**2.5**	**0.11**	**0.15**	**1.2**	**0.11**	**0.18**	**>100**
**Z23**	**1,000**	**1,100**	**31,500**	**14,000**	**6,500**	**1,700**	**2,500**	**1,000**	**7,100**	**2,200**	**1,000**	**5,600**	**90**

The numbers correspond to the reciprocal of the dilution of the guinea pig sera at which the luciferase expression is reduced by 40% (IC_40_). One negative control is the related but non-HIV-1 pseudovirus, amphotropic murine leukemia virus (α-MLV). Another negative control is HRV14 without any of the HIV MPER inserts. The positive control is serum from an HIV-infected person; guinea pig titers are expected to be lower as HIV does not replicate in guinea pigs.

The C1-8 chimeric virus failed to generate detectable neutralization activity against any of the isolates examined. In contrast, the B1-16 chimera elicited neutralizing activity for all three guinea pigs tested. The greatest response was seen in the sera’s ability to neutralize the 92TH024 pseudovirus (of subtype AE; co-receptor type R5), with IC_40_ reciprocal neutralizing titers of 70, 80, and 80. The 93BR029 pseudovirus (of subtype F; co-receptor type R5) was neutralized with IC_40_ titers of 30, 30, and 50. The 92UG005 pseudovirus (of subtype D; co-receptor type R5) was neutralized by one of the guinea pig sera with an IC_40_ titer of 80.

### Construction and Production of the Three Combinatorial 4E10 Libraries

#### 4E10 Library I

A complex library was constructed to encode both the 2F5 and 4E10 epitopes in tandem in place of part of the VP2 puff of the NIm-II region of HRV14 (4E10 Lib I, Figure 1). The 2F5 and 4E10 epitopes are naturally adjacent in the context of HIV-1, offering the hope that their joint inclusion might enhance our ability to present them in native-like conformations and to maximize the opportunities for eliciting an effective immune response. The 4E10 epitope consists largely of the tryptophan-rich, contiguous sequence N^671^WF(D/N) ITNWLW^680^ [33,62,63,64,65] and is recognized by the 4E10 mAb, one of the most broadly neutralizing anti-HIV antibodies, capable of neutralizing virus isolates from all group M HIV-1 subtypes [21,63,66,67]. Crystallographic and NMR studies have demonstrated that the 4E10 epitope adopts an amphipathic helical conformation, significantly embedded in the viral membrane and varying in the extent to which it is slightly bent or straight in the middle of the epitope, depending on the constructs and conditions used [29,33,57,65,68]. The binding of mAb 4E10 to the epitope appears to create a more significant angle at the bend between the two α-helical segments of the MPER epitope region [29], initiated when the CDR H3 loop extracts W672 and F673 from the viral membrane, promoting neutralization. The 4E10 epitope overlaps with that of the Z13e1 epitope, W^670^NWFDITN^677^ [69]. The Z13e1 epitope consists of a two-helix structure with a hinge near D674, which distinctly kinks and rigidifies upon binding by Z13e1 [28,57,29].

For the 2F5 epitope, the (E60/A40) LDKWA sequence was chosen, reflecting the approximate serodiversity of E (60%) and A (40%) residues in the Los Alamos Database at the time of the design. For the 4E10 epitope, a more complex sequence was chosen than those used for the 2F5 chimeras, reflecting the more diverse sequences found in the Los Alamos Database as well as our attempt to mitigate some of the extremely hydrophobic nature of the epitope (to avoid problems with its presentation on the solvent-exposed surface of HRV14). We chose the following sequences (with the subscripts representing the percentages of the residues found at any given site for any cases where its value was less than 100%):


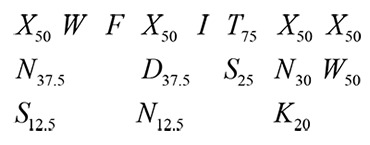


We represented the 1-3 key residues found at any given site for this epitope and, in all cases except those that appeared to be both invariant and critical to interaction with 4E10, we allowed for 50% randomization of the residue (X, encoding any of the 20 amino acids, with the remaining percent reflecting the natural ratios of the given residues seen among HIV-1 isolates). The same reasoning was used to encode the three residues between the epitopes. A mixture of HIV residue sequences and randomized sequences was also used for the linkers that flanked the tandem epitope, with one added degree of complexity: variability of the length of the linkers. These linkers varied from 1–4 residues in length, which provided additional chances for getting the orientation of key residues appropriately presented. Unfortunately, we were unable to obtain any chimeric viruses that displayed both epitopes.

#### 4E10 Library II

A second 4E10 combinatorial library was constructed that was similar in design to Library I but without the 2F5 epitope (4E10 Lib II, Figure 1). The 4E10 epitope chosen corresponded to the same residues except we replaced the C-terminal W with an A, removed the randomization of the epitope residues, and adjusted a few percentages of the HIV residues to confer a slightly more hydrophilic balance. In addition, instead of encoding 1-4 linker residues, the epitope was flanked by 1, 2, 4, or 6 residues (roughly half of which were randomized and half of which represented the key HIV-1 residues found at each site).

Anticipating possible viability problems, this library was constructed in 16 subpools, by separately combining each of the four N-terminal DNA oligomers with each of the four C-terminal DNA oligomers. For most of the subpools, no viable chimeric viruses were obtained, particularly for the subpools designed with longer oligomers. For the chimeric viruses that were obtained, the corresponding plasmid sequences were all found to be intact; however, the transcribed viral RNAs were typically characterized by multiple point mutations and deletions (data not shown). In fact, the only chimeric viruses found to have intact or nearly intact viral RNA sequences were those that had an N-linker length of one and a C-linker length of either one or four (both of which could present an α-helical structure in the same register).

#### 4E10 Library III

**Figure 4:**
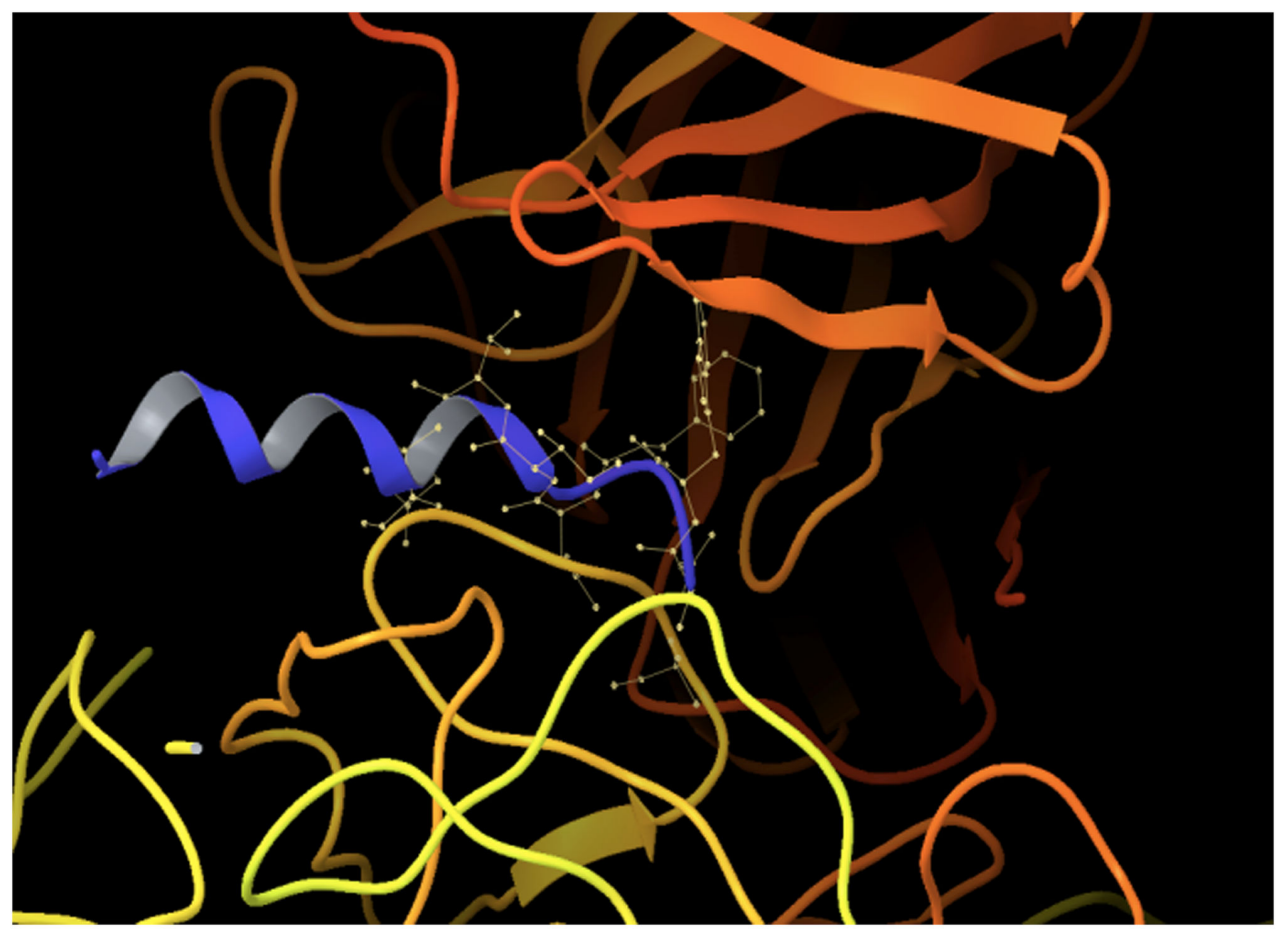
Immunoselection scheme used with 2F5 Library **I**. The 2F5 Library I was subjected to selection using varying amounts of competitive peptide Ac-EQELLELDKWASSLW-NH_2_ in hopes of finding chimeras that were well recognized by 2F5. The chimeras used for immunization studies were subjected to a single round of immunoselection.

A third 4E10 combinatorial library was constructed by trimming the epitope down to its most essential components (including the N671 and D674 residues critical for neutralization by Z13e1 [69] to allow for the presentation of either or both epitopes; Figure 1). With known X-ray crystallographic structures of the 4E10 Fab bound to a number of cognate epitope peptides, we juxtaposed the LWNWFDITNW 4E10-peptide complex structure (PDB ID 2FX7) with that of the VP2 puff region of HRV14 (Figure 4) to design a third library aimed at conserving the integrity of the structure of both the HIV epitope and HRV. With the goal of inserting an HIV epitope with linkers of optimal length to “fit” in place of the HRV loop and promote the helical conformation of the 4E10 peptide seen crystallographically, we chose the immunogenic sequence, NWFDIT, flanked on the C-terminal side of the epitope by the prevalent N or K residues (25% prevalence each, as well as 50% randomized residues), with an additional 1-3 linker residues on each side of the epitope. On the N-terminal side of the epitope, half of the linker residues were randomized and half were coded to be the α-helix-promoting A and E residues and the more hydrophilic bend- and turn-promoting D (Figure 1).

After sequencing the viral RNA, the intact 4E10 epitope (NWFDIT) was observed in 16 of 33 chimeric viruses (found in 11 of the 16 sub-libraries examined; unpublished data). Among the 17 mutated viruses, all but one involved mutation of the strongly hydrophobic tryptophan residue, indicating that the hydrophobicity of this residue was detrimental to the viability of the chimeras. As with 4E10 Library II, most of the frame-shift mutations were observed among chimeras with the greatest number of linker residues, suggesting that longer linkers led to reduced viral fitness. When analyzing the linker residues of the viruses with intact 4E10 epitopes, the linker residues were found to be more abundant on the C-terminal side of the epitope, with an average of 1.7 vs. 1 residues/linker (for the C vs. N linker). For the biased residues encoded to be D, A, and E, E was favored [representing 20/38 (57%) of the D/A/E biased residues vs. an expected percentage of 33%]. Among the randomized residues, there was a preponderance of G residues in the N-linkers, particularly for the chimeras with longer linkers, suggesting that the G residues may have been serving a role in disrupting the secondary structures that would have otherwise characterized these linkers.

### Immunoselection of Library III

**Figure 5:**
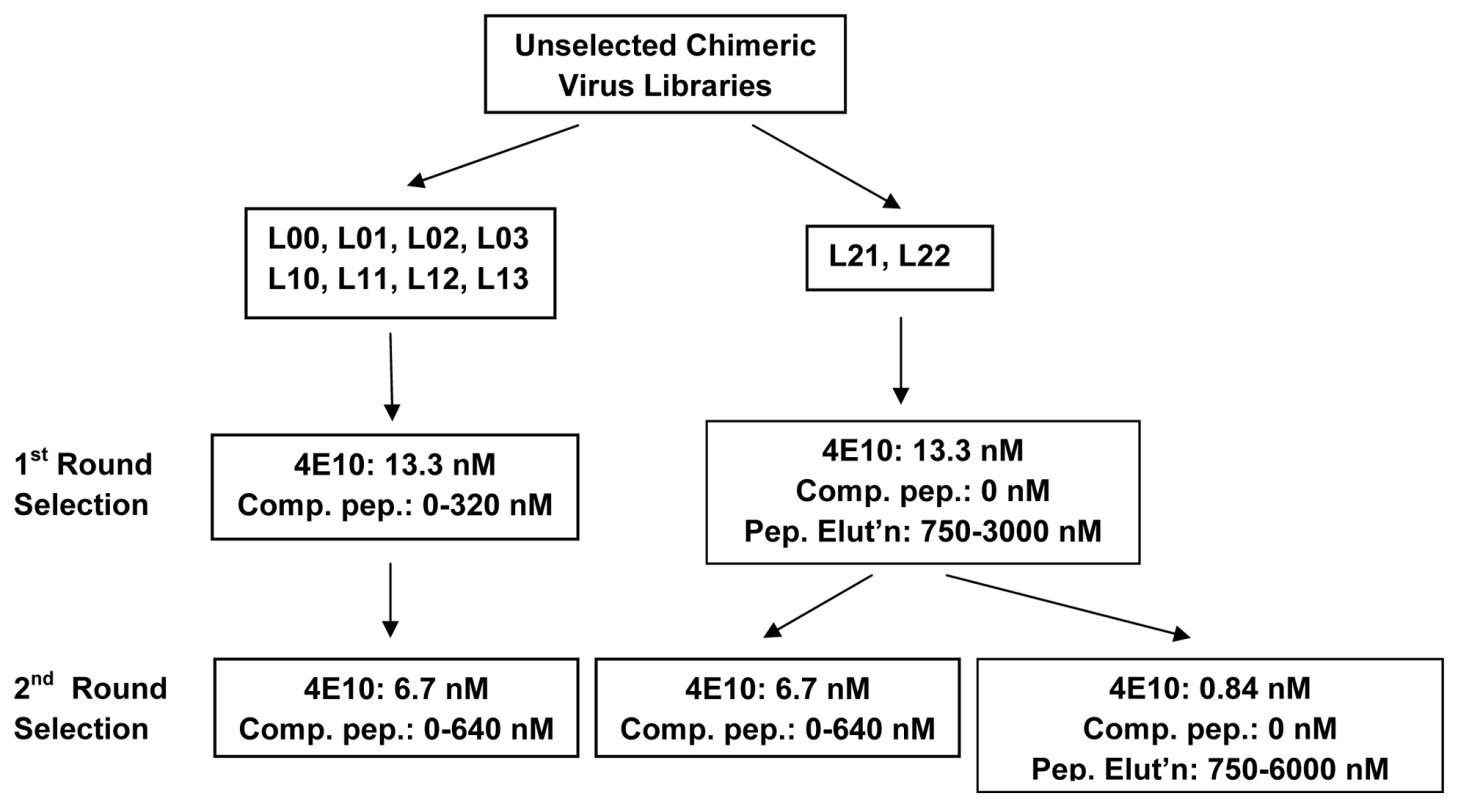
Immunoselection scheme used with 4E10 Library III. The sub-libraries were subjected to varying selection schemes in hopes of finding chimeras that were well recognized by 4E10. All of the chimeras used for immunization studies were subjected to two rounds of immunoselection, some in the presence of competitive peptide NK-15.

To enrich for the most immunogenic chimeric isolates, the 11 (of 16) individual combinatorial sub-libraries seen to have chimeras with mostly or completely intact 4E10 epitopes were subjected to immunoselection (Figure 5). Initial experiments were performed with both the 4E10 and Z13e1 IgGs, but we were unable to obtain specific and selective binding with either of two sublibraries tested using Z13e1 (L12 and L13); therefore, subsequent immunoselections were all performed with 4E10 alone. After one of the individual sub-libraries, L20, showed poor antigenicity (i.e., poor ability to bind 4E10) after the first round of immunoselection, it was eliminated from further immunoselection studies, leaving 10 sub-libraries that were subjected to two rounds of immunoselection.

Two different immunoselection schemes were employed for the 10 libraries (Figure 5). For libraries L00, L01, L02, L03, L10, L11, L12, and L13, two rounds of competitive immunoselections were used. In the first round of competitive immunoselection, 13.3 nM of 4E10 antibody was coated on the immunoplates, and then 0-320 nM of the 4E10-epitope peptide [designated NK-15 (with the helix-promoting K7-D11 lactam-bridged sequence Ac-NWFDITK _7_WLWD _11_KKK-NH_2_)] was used to compete with the input library viruses for binding to the 4E10 antibody. After washing away unbound material, H1-HeLa cells were seeded in the wells and the resulting subpools of viruses harvested were collected and characterized by direct ELISA. In each case, the subpool that bound 4E10 with the highest average affinity was used for the second round of immunoselection. The second round of immunoselection was more stringent, employing 6.7 nM of 4E10 antibody and 0-640 nM peptide. In contrast, libraries L21 and L22 were not subjected to competitive binding of virus to antibody. After these chimeras were allowed to bind to 13.3 nM 4E10, 750-3000 nM NK-15 peptide was used to elute the bound viruses (prior to cellular elution). Differentially eluted subpools were collected and characterized. The subpools with the greatest average affinity to 4E10 were followed by further immunoselection using either 6.7 nM 4E10 and 0-640 nM competitive peptide or 0.84 nM 4E10, no competitive peptide, and 75-6000 nM peptide for elution.

**Figure 6:**
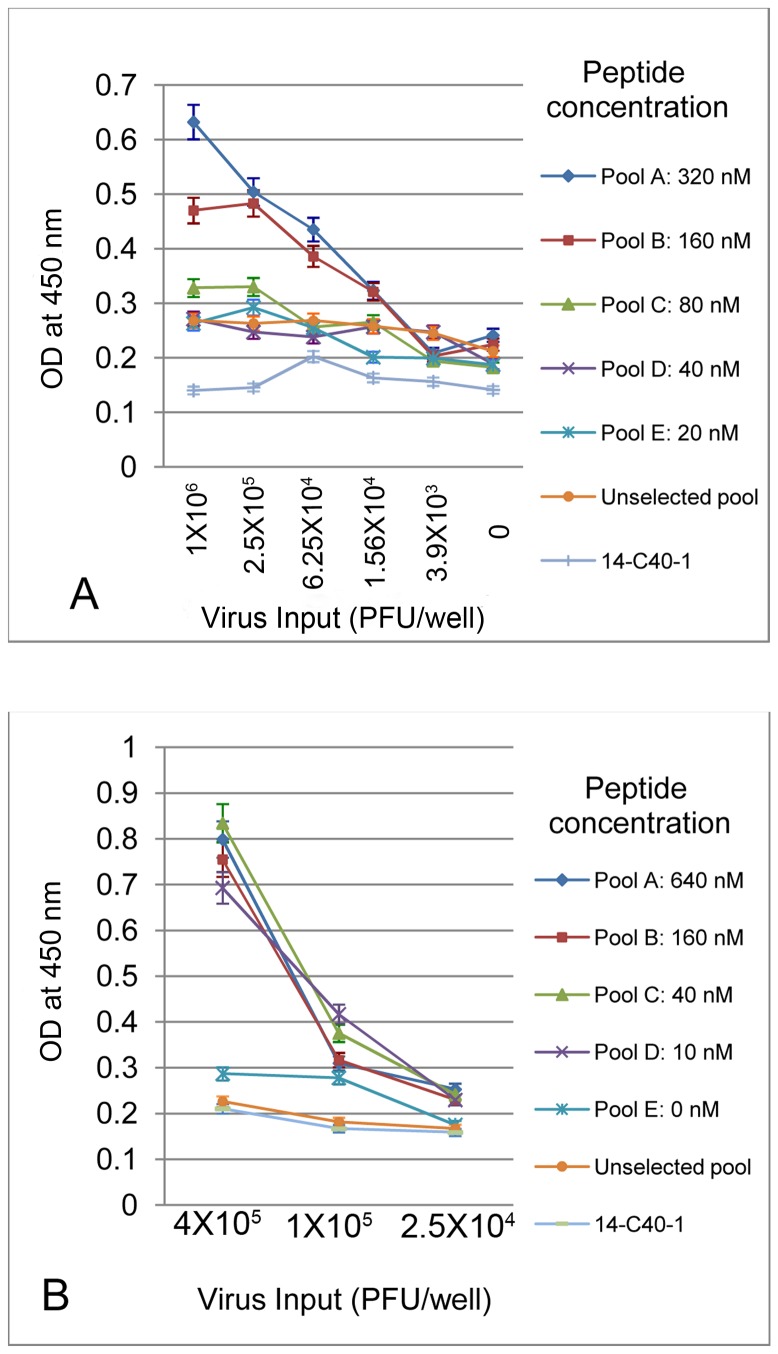
Direct ELISAs reflecting the impact of immunoselection of 4E10 library III, sub-library 13. Immunoselection was performed by immobilizing 4E10 and capturing chimeras that could bind in the presence of varying amounts of competing 4E10 peptide. **A**. 1st round: Immobilized 4E10 mAb treated with library 13 plus competitive peptide. **B**. 2nd round: Immobilized 4E10 mAb treated with Pool A from 1st round plus competitive peptide.

To evaluate the effects of immunoselection on the enriching of chimeric viruses with enhanced 4E10 binding affinity, direct ELISAs were performed. Figure 6 shows an example of two rounds of immunoenrichment with library L13. The ELISA reflects that the binding affinity varies significantly for viruses from different subpools, with the highest concentrations of competitive peptide used during immunoselection being associated with greater average binding affinity of the viruses to immobilized 4E10 (Figure 6A). In contrast, a 2F5 chimeric virus, 14-C40-1 (which expresses the 2F5 epitope in lieu of the 4E10 epitope), was not influenced by the 4E10 peptide concentration. It appears that there was no further immunoselection achieved in the second round (Figure 6B), reflected by the lack of significant differences of the OD_450_ values for the 10-640 nM peptide-selected subpools.

### Antigenic characteristics of immunoselected chimeric viruses from Library III

After two rounds of immunoselection, viral plaques were isolated from each immunoselected virus pool. The virus isolates were subjected to two rounds of plaque purification and then propagated. Propagated virus lysates were assayed using direct ELISAs to characterize their antigenicity. In a preliminary ELISA, 30 virus samples were compared. The 10 viral isolates that were found to bind 4E10 most tightly were further propagated, partially purified, and subjected to additional characterization by direct ELISA, competitive ELISA, and MTT neutralization assays.

**Figure 7:**
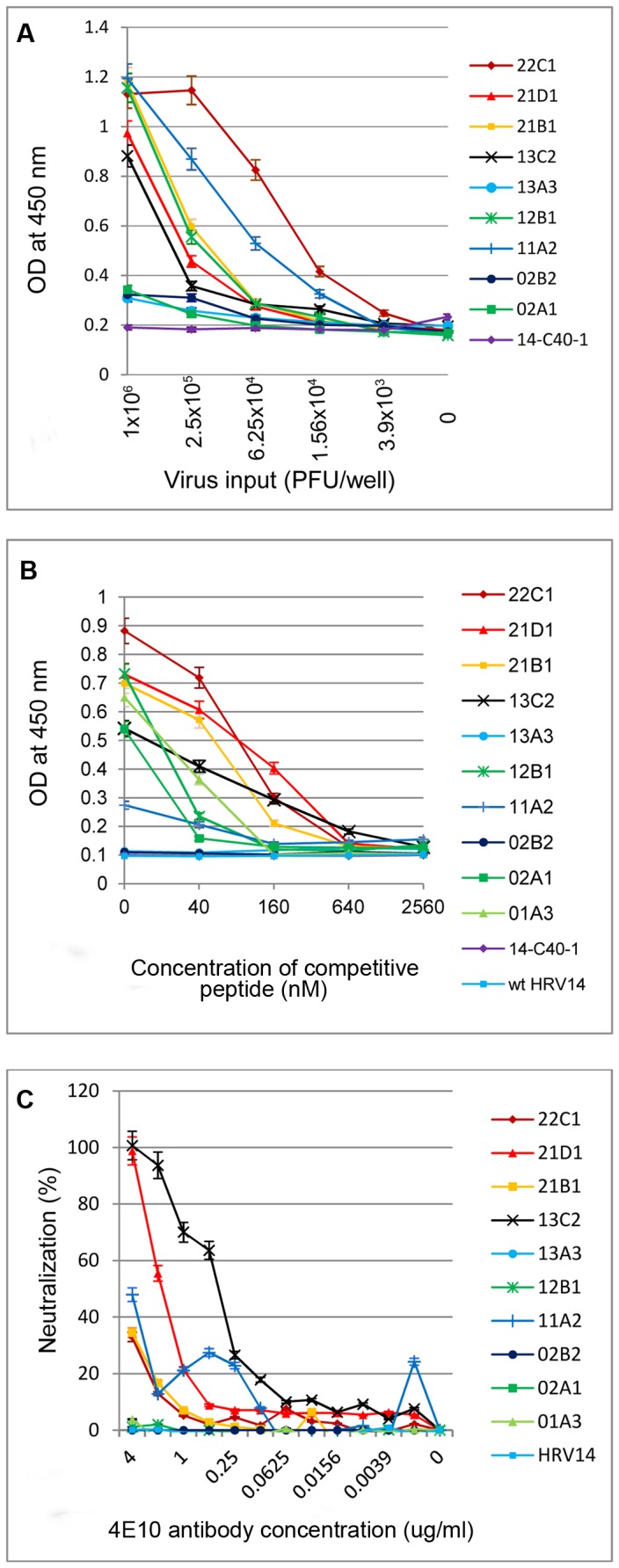
Antigenicity assays reflecting differences among individual chimeric viruses. **A**. Direct ELISA using 4E10 to capture the chimeras. **B**. Competitive ELISA using 4E10 to capture chimeras that could bind in the presence of competitive peptide, NK-15. **C**. MTT cell-killing assay using 4E10 to bind to and neutralize the chimeric viruses, preventing HeLa cell death.

Direct ELISAs, performed with 9 of the 10 chimeras chosen for further study, showed significant antibody binding for 6 of the viruses tested (cf. 22C1, 11A2, 21B1, 12B1, 21D1, and 13C2; Figure 7A). The others (13A3, 02B2, and 02A1) bound 4E10 with affinity barely greater than that of the 14-C40-1 chimera containing the 2F5 epitope in lieu of the 4E10 epitope, indicating that they most likely had bound 4E10 in the immunoselections predominantly via non-specific hydrophobic interactions. It appears to be significant that two of the three poor 4E10 binders lack N-terminal linkers altogether and that the third chimera has helix-disrupting P residues on both sides of the core epitope.

Using a competitive ELISA format, we tested the ability the 10 chimeric viruses that bound 4E10 best in the immunoselections to compete with the constrained 4E10 epitope peptide, NK-15, for binding to the 4E10 antibody. The observations paralleled those of the direct ELISA (Figure 7B). Five of six of the viruses with the greatest affinity to 4E10 in the presence of competitive 4E10 peptide were the same as those found using the direct ELISAs without the peptide (e.g., 21D1, 22C1, 21B1, 13C2, and 12B1, but not 11A2). It may be noteworthy that the sixth chimera, 11A2, which bound poorly in the competitive ELISA chimera, is the only chimera that bound in the apparently less stringent direct ELISA that has only a secondary-structure-disrupting S residue in its N-terminal linker (and may not be able to accommodate possible structural requirements for optimal 4E10 binding).

MTT neutralization assays were also employed to characterize the antigenicity of the 10 chimeric virus isolates (Figure 7C). In this case, we asked if 4E10 could recognize the transplanted HIV epitope, now on the heterologous virus, HRV14, and in so doing, neutralize the ability of the chimeric HRV to infect its host cells, H1-HeLa cells. While this test did not point to the same 5 or 6 viruses as the ELISAs, two of the six viruses, 21D1 and 13C2, stood out as being clearly recognized and neutralized by 4E10 in a dose-dependent manner. In addition, 4E10 did display some mild neutralization of three of the other four chimeras that had scored well in the ELISAs, 11A2, 21B1 and 22C1, further verifying that some aspects of the antigenicity of the 4E10 epitope in the context of these five chimeric viruses was recognized by 4E10 in the context of HRV.

### Sequence analysis of the immunoselected chimeric viruses from Library III

The sequences of the foreign epitopes of the 10 4E10 epitope-containing chimeric viruses that best bound 4E10 in the three antigenicity assays were determined (Table 3). Unlike the chimeric viruses of Libraries I and II, all 10 of the chimeras sequenced from Library III contained full-length, unmutated 4E10 core epitope sequences. However, a non-random distribution of residues was found in both linker regions.

**Table 3:** Sequences of the most antigenic chimeras immunoselected and characterized from 4E10 Library III.

**Chimeric viruses**	**HRV**	**N linker**	**Core epitope**	**C linker**		**HRV**
**4E10 Library III Chimeras**						
**01A3**	… DLSS		NWFDIT	N	E	VGG…
**02A1**	… DLSS		NWFDIT	K	EE	VGG…
**02B2**	… DLSS		NWFDIT	K	LE	VGG…
**11A2**	… DLSS	S	NWFDIT	N	E	VGG…
**12B1**	… DLSS	P	NWFDIT	K	EP	VGG…
**13A3**	… DLSS	E	NWFDIT	N	EAN	VGG…
**13C2**	… DLSS	E	NWFDIT	N	NLV	VGG…
**21B1**	… DLSS	DD	NWFDIT	N	T	VGG…
**21D1**	… DLSS	SD	NWFDIT	N	E	VGG…
**22C1**	… DLSS	DE	NWFDIT	K	N	VGG…

Most chimeras isolated from the 4E10 libraries I and II were found to have deletions or mutations in their inserted sequences. Most chimeras from Library III had correct sequences (matching the design). This set represents those determined to have the most promising antigenic characteristics and, therefore, chosen for immunization studies in guinea pigs.

The N-terminal linker was expected to consist of 1-3 residues, 50% of which were randomized and 50% of which were equally distributed among D, A, and E. Surprisingly, the linkers were found to have 0-2 residues instead (despite having 1-3 residues in the sequenced plasmids). Furthermore, the N-terminal linkers were populated with more E and D residues than expected (accounting for 7 of the 10 residues vs. 4 of 10 expected), generating an observed average charge per linker of -0.7 (vs. -0.33 expected; this is in contrast to their pre-immunoselected counterparts, which had normal charge distributions of the N-linker residues). The E residues (3 of the 10 residues) strongly promote α-helices and D residues (4 of the 10 residues) are known to promote β turns. The remaining three N-terminal linker residues consisted of two S residues and one P residue, both of which are known to disrupt secondary structures and promote β turns. The propensities of the N-terminal linker residues to promote α-helices were similar for the selected and unselected chimeras from 16 subpools pre-immunoselection (18 of which were mutated in the NWFDIT epitope region; Table S1). The immunoselected chimeras exhibited 30% α-helix-promoting residues vs. 38.7% observed for the unselected chimeras. It is interesting to note that both the selected and unselected chimeras had fewer α-helix-promoting N-linker residues than was expected from the nucleotide sequences used (53.1%), most likely reflecting a cost to fitness of the chimeric viruses. In contrast, the propensity of the N-terminal linker residues to promote β turns was significantly increased by immunoselection [promoted by 7/10 residues (70.0%) vs. 26% expected]. In addition, like the N-terminal linker residues of the selected viruses, those of the unselected viruses were shorter on average than expected (with only 2 of 33 chimeras having N linkers with 3 residues in length).

The C-terminal linkers of the 10 immunoselected chimeras that best bound 4E10 were found to be of the expected lengths (with a modest preference for shorter linkers); however, their sequences were non-random in nature. At the position C-terminal to the core epitope sequence, designed to encode 50% randomized residues and 25% each of the HIV-1 residues at that site, N (an α-helix and β-sheet breaker) and K (an α-helix promoter), only N and K were found. Unfortunately, this observation was also found for 20 of the 25 intact unselected chimeras and even for 9 of 11 plasmid DNAs.

C-terminal to the X/N/K residue, the chimeras were designed to have their linker residues 50% randomized and 50% (D/A/E). The observed average charge of the C-terminal linker residues was -0.47 (vs. -0.33 expected). In addition, the amino acid distribution was non-random (Table S1), reflecting fitness needs for epitope presentation on HRV (in the case of unselected chimeras) and attributes that favored binding to 4E10 (in the case of the immunoselected chimeras). Oddly, there were no bend- and turn-promoting D residues observed among the immunoselected viruses (vs. 3-4 expected). Instead, 12 of the 17 linker residues (70.6%; Table S1) were α-helix-promoters (8 E, 2 L, 1 A, and 1 V) and 12 (70.6%) were β-sheet breakers (8 E, 1 P, and 1 T). Whether immunoselected or not, the C-terminal linker residues displayed slightly more negative charge than expected (-0.44 per linker vs. -0.33 expected). Regarding the amino acid distribution, immunoselection led to increases in the presence of C-terminal linker residues that promoted α-helix formation, β-sheet disruption, and β-turn formation. α-helix formation was promoted by 70.6% of the selected linker residues vs. 60.5% for the unselected set and 53.1% expected at the nucleotide level. β-sheet disruption was also promoted by 70.6% of the selected linker residues vs. 52.6% for the unselected set and 25.0% expected at the nucleotide level. β-turn formation was promoted by 40% of the selected linker residues vs. 31.6% for the unselected set and 26.6% expected at the nucleotide level.

### Physical appearance of isolated chimeric viruses from Library III

**Figure 8:**
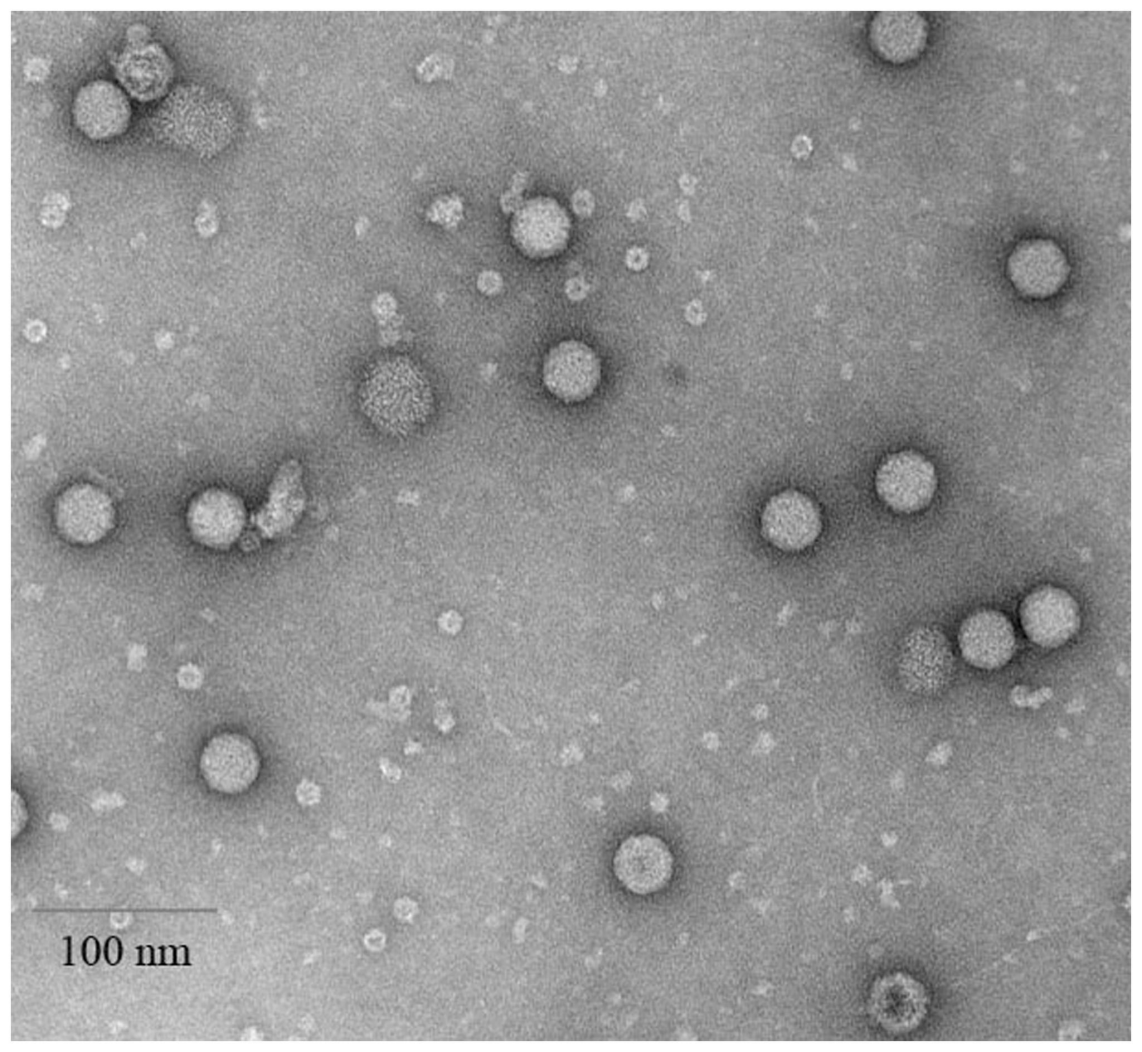
Transmission electron micrograph of a representative partially purified preparation of chimeric virus 11A2. Intact viruses are roughly 30 nm in diameter. Pentameric units, 12 of which come together to form the virus coat, can be seen as small discs roughly 5 nm in diameter (with dark stain-filled centers), as can some smaller fragments, indicating that some viruses are not fully assembled.

Transmission electron microscopy was performed with each of the chimeric viruses chosen for immunization studies (one of which is exemplified in Figure 8). All preparations of mini-purified viruses were seen to include a noticeable fraction of viruses that were not fully assembled, as evidenced by the presence of pentameric subunits (12 of which come together normally to form the intact virus coat) uncharacteristic of wild-type HRV14. In addition to electron microscopy, dynamic light scattering revealed that the chimeric viruses studied were monodisperse in the concentration ranges used (unpublished data).

### Immunization of guinea pigs and characterization of immunogenicity of Library III chimeric viruses

To characterize the ability of the chosen 10 chimeric viruses to elicit 4E10-like antibodies, we immunized guinea pigs (three per chimera) using a prime-boost strategy. Immunizations consisted of subcutaneous injections of 40 µg of mini-purified virus at weeks 0 and 4 followed by immunizations at weeks 9 and 13 with 40 µg of mini-purified chimeric virus boosted with 80 µg of the KLH-conjugated lactam-bridged 4E10 epitope peptide, NK-15, shown to bind 4E10 with the greatest affinity from a series of peptides tested [70]. Sera were collected three weeks later after which their neutralizing abilities were tested against 12 HIV-1 pseudoviruses that contained envelope sequences from diverse subtypes (A, B, C, D, AE, and F) and co-receptor usages (X4 and R5).

**Figure 9:**
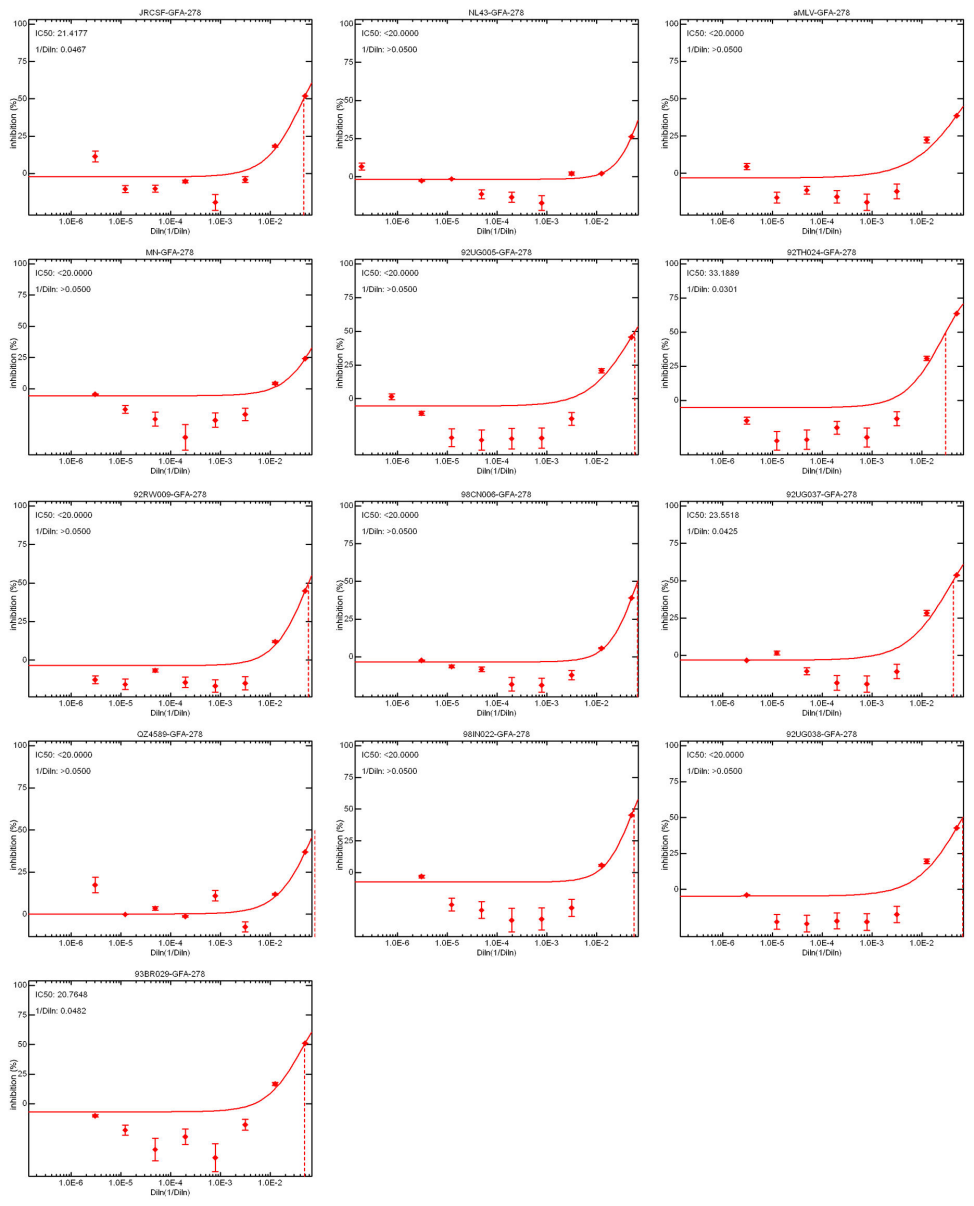
Representative neutralization curves reflecting the ability of antiserum GFA-278, derived from immunization with chimeric virus 12B1, to neutralize 12 HIV pseudoviruses from six subtypes. Neutralizing titers corresponding to 50% inhibition of the reporter luciferase activity (compared with inhibition by pooled normal guinea pig serum values) is denoted by the dashed lines.

The data were generated as neutralization curves, exemplified by the Figure 9 curves obtained with guinea pig serum GFA-278 (from immunization with chimeric virus 12B1). The neutralization titers were expressed as reciprocal titers at which 40% inhibition of neutralization was observed compared with pooled normal guinea pig serum values. Only a few serum samples were able to generate 50% inhibition of infection by the HIV‑1 pseudoviruses, so the less stringent cut-off of 40% inhibition was chosen to help identify and differentiate chimeras that could be further developed for possible use as vaccine components. Given that human rhinovirus does not replicate in guinea pigs, but does replicate in humans, it is possible that the immune responses to the chimeras would be greater in humans.

Table 2 shows the overall neutralization activity of the 30 serum samples elicited from the chimeric viruses as well as pooled serum from six guinea pigs immunized with a non-engineered HRV14, serum from HIV-1-positive patient Z23, and purified mAb 2F5 and 4E10 IgGs. The non-HIV-1 pseudovirus, αMLV, was used as a negative control. We have previously immunized guinea pigs with KLH-conjugated MPER peptides alone, resulting in no significant neutralizing responses [35]. Likewise, the lack of significant neutralization elicited by the majority of the animals immunized in this study (Table 2) further reflects that the peptides used were not able to elicit neutralizing responses on their own. Whether or not the peptides provided a beneficial boosting of the immunity elicited by the immunogenic chimeric viruses studied is not known, as peptides were added for every immunization in this study.

As expected, significant differences can be seen comparing the abilities of the chimeras to elicit neutralizing responses not only among the different pseudoviruses, but also among the different guinea pigs. The broadest neutralization of the pseudoviruses was elicited by the serum sample GFA-278, from chimera 12B1, which neutralized 10 of the 12 HIV pseudoisolates, including the two subtype C pseudoviruses (which are moderately neutralized by purified mAb 4E10 but are not neutralized by mAb 2F5 [63]). At least one pseudovirus of each subtype and co-receptor usage tested was neutralized by serum from this chimera, with reciprocal titers ranging from 30 to 80. The other sera elicited by the 12B1 chimera, GFA-277 and GFA-279 were not as inhibitory. GFA-277 only neutralized one isolate, 92TH024 (subtype AE, with a rather low titer of 30). GFA-279 neutralized four pseudoviruses of four subtypes (92UG037 of subtype A, JRCSF of subtype B, 92TH024 of subtype AE, and 93BR029 of subtype F, with titers ranging from 30–90). The αMLV pseudovirus was also neutralized marginally by this serum sample (GFA-279), suggesting that there may have been some degree of non-specific neutralization activity by this serum. Notably, the 12B1 chimera was not among those best recognized by 4E10 in the ELISAs (Figures 6 and 7), making it difficult to understand why this serum displayed the broadest—and some of the strongest—neutralizing ability. The GFA-265 serum sample elicited by chimera 22D1 was also able to modestly neutralize six pseudoviruses; however, this serum also had a comparable neutralizing titer of 40 against αMLV, and the other two sera from immunization with this chimera were inactive, suggesting that the neutralizing activity of this serum is not likely to be significant. In contrast, the neutralization spectrum seen in response to 22C1 demonstrated specific and reproducible neutralization of several pseudoviruses. All three guinea pig sera neutralized 93BR029 (subtype F, with IC_40_ values of 85, 70, and 25) and two serum samples neutralized 92TH024 (subtype AE, with IC_40_ values of 50 and 25). In addition, GFA-261 was also able to neutralize 92UG005 (subtype D) and JRCSF (subtype B), both with a titer of 45. A spurious titer was exhibited (repeatedly) for the GFA-276 serum elicited by chimeric virus 13A3, registering 875 against the subtype B NL43 pseudovirus; no other neutralizing activity was seen against any of the other pseudoviruses for any of the three serum samples against this chimera. Modest neutralization responses were seen against the 02A1 and 02B2 chimeras. Both elicited neutralization activity in two of three guinea pigs, generally against the same pseudoviruses, most strongly against 92TH024 (subtype AE, ranging from 35–60 in four of six guinea pigs), 93BR029 (subtype F, with titers of 35 for two of six guinea pigs) and 92UG005 (subtype D, with titers of 35 and 25 for two of six guinea pigs). Overall, the pseudoviruses most frequently neutralized by the various serum samples were 92TH024 (subtype AE, also observed with a number of 2F5 chimeras [35]) and 93BR029 (subtype F), though the reason for this is not clear.

## Discussion

During natural infection, non-neutralizing antibodies appear to be dominant in the antibody response directed against HIV-1 gp41 [71,72,73,74]. Using the chimeric HRV system to display the MPER epitopes that elicited the broadly neutralizing mAbs, 2F5, 4E10, and Z13e1, we have attempted to focus the immune response specifically to these conserved epitopes. Many attempts have been made with little success using the ELDKWA (2F5) epitope as a vaccine target (reviewed in Table S1 of [35]). Similarly, many efforts to display the 4E10 epitope have also failed to induce neutralizing antibodies [64,75]. Unfortunately, significant MPER-directed neutralization has been limited [15,35,36,37,38,39]. It is likely that the vaccine candidates tested thus far do not sufficiently resemble the HIV epitopes in their most immunogenic conformations.

In our previous studies [35,42], we engineered the 2F5 epitope based on structural considerations and constructed a series of combinatorial ELDKWA libraries comprising millions of chimeric viruses with different structures. Following immunoselection with 2F5, we were able to elicit neutralizing antibodies in animal models. For this study, armed with additional structural information, we designed new presentations of the 2F5 epitope. We aimed to present the 2F5 epitope to be well exposed, and with the addition of the flanking N- and C-terminal hydrophobic L residues that were shown to increase peptide binding to 2F5 by three orders of magnitude [61]. Our previous efforts to present the flanking L residues were unsuccessful in the context of HRV [35] and, in this study, the presence of the flanking L residues was associated with neutralizing titers that were less broad and, in some cases, less potent than those obtained from our prior efforts. Nonetheless, one of the two ELDKWA-presenting chimeras was able to elicit neutralizing antisera in all three guinea pigs tested. Two of the antisera were able to neutralize pseudoviruses of two subtypes and the third antiserum was able to neutralize pseudoviruses of three subtypes.

We also used a structure-based strategy for presenting the 4E10 epitope. In this case, the designs included the essential HIV residues and also the goal of maintaining the α-helix conformation recognized by 4E10, all using the combinatorial approach characteristic of our previous efforts (to increase the chances of generating the desired structures) combined with the high-throughput screening. This semi-rational, brute-force effort resulted in the production of three 4E10-epitope-displaying libraries. The first two such libraries were met with viability challenges and/or deletions in the region of the foreign epitope. The third library was more successful and most individual chimeras examined retained the intact epitope and demonstrated high specificity of binding to 4E10, particularly following immunoselection. In contrast, attempts to immunoselect chimeras with the Z13e1 antibody did not lead to specific binding, demonstrating a distinction between the epitopes of Z13e1 and 4E10.

Sequence analysis revealed significant effects of virus viability and antibody-binding capabilities upon the distribution of linker residues found flanking the 4E10 epitope (Table 3 and Table S1). In the N-terminal linker, the percentage of α-helix-promoting residues found was lower than expected among unselected chimeras (38.7% vs. 53.1% encoded) and reduced further following immunoselection with 4E10 (to 30.0%). In contrast, the percentage of β-turn promoting residues was strikingly increased as a function of being presented on the surface of HRV (48.4% vs. 26.6% encoded), and this was even more dramatic following immunoselection (70.0%). Despite the reduction in α-helix-promoting residues in the N-terminal linker, the net result was that the percentage of chimeras with either α-helix- or β-turn promoting residues in their N linkers was 100% for the immunoselected chimeras, in contrast to being 80.6% for the unselected chimeras.

At the C-terminal linker, beyond the N- and K-biased linker residue, immunoselection led to a significantly greater fraction of α-helix-promoting residues, β-sheet-disrupting residues, and β-turn-promoting residues (Table S1). The percentages of α-helix-promoting residues increased from 60.5% among unselected chimeras to 70.6% for immunoslected chimeras (vs. 53.1% encoded). Likewise, the percentages of β-sheet-disrupting residues increased from 52.6% among unselected chimeras to 70.6% for immunoselected chimeras (vs. 25.0% encoded). Similarly, the percentages of β-turn promoting residues increased from 31.6% among unselected chimeras to 40.0% for immunoselected chimeras (with 26.6% encoded).

The N and C linker distributions of the 4E10 chimeras clearly reflect crucial roles of the residues present. Regarding viral fitness, benefits were seen on (a) the N-terminal side from reductions in the percentages of α-helix-promoting residues and increases in the percentages of β turn-promoting residues and (b) the C-terminal side from the presence of α-helix-promoting residues, β-sheet-disrupting residues, and β-turn promoting residues. Regarding binding of the engineered epitope to 4E10, benefits were seen on (a) the N-terminal side from the presence of even greater reductions in the percentages of α-helix-promoting residues and greater increases in the percentages of β-turn-promoting residues and (b) the C-terminal side from increases in the α-helix-promoting residues, β-sheet-disrupting residues, and β-turn promoting residues. While we can only conjecture about these amino acid distributions, it is interesting to note that: (1) β-turns are typically the dominant secondary structure of antibody-bound peptides [76] and (2) it may be that β turns in these chimeras enabled the 4E10 epitope to maintain the α-helicity that appears to be necessary for 4E10 binding [64,65]. In this regard, it may be significant that the most broadly immunogenic chimeric virus identified by this work, 12B1, has short, strongly turn-promoting linker residues, with P residues on both sides of the epitope.

The antigenicity of the chimeric viruses (i.e., the ability of the viruses to bind to 2F5 or 4E10) was evaluated using direct ELISAs, competitive ELISAs, and an MTT cell-killing assay. It is worth noting that the ELISAs required considerable optimization to overcome complications associated with the extreme hydrophobicity of the MPER region (particularly for the 4E10 epitope) as well as of the antibodies (particularly 4E10). Indeed, the CDR H3 of 4E10 is extraordinarily hydrophobic and polyspecific [77,78], requiring optimal choices for the preparation of the reagents and selection of solvents. The 4E10 peptide, in particular, was rendered soluble by the inclusion of an α-helix-promoting lactam bridge between the K7 and D11 of the peptide, a series of solubilizing lysines at the C-terminus, and dissolution in 10% acetic acid. The results obtained from the different binding assays were highly concordant. Overall, the binding assays revealed that the chimeric virus with the longer linkers and greater percentage of α-helix promoting residues displayed the more favorable binding to 4E10.

Using a prime-boost approach for immunization, guinea pigs were primed with chimeric viruses and boosted with chimeric viruses and KLH-conjugated epitope peptides. The sera generated from the guinea pigs were then tested for their ability to neutralize HIV-1. Numerous sera elicited from a variety of the chimeric viruses showed a notable breadth of their ability to neutralize varied HIV pseudoviruses. It is worth noting, however, that the neutralization was modest, predictably as impacted by the choice of the MPER target, which has yet to elicit high titer neutralizing responses in any recombinant setting, and also by the fact that HRV does not replicate in guinea pigs. In this regard, any neutralization of HIV elicited by an MPER-based construct constitutes a notable achievement.

Remarkably, one chimeric virus, 12B1, generated an exceedingly broad neutralization spectrum. Serum from this chimera was able to neutralize 10 of 12 pseudovirus isolates. Four other chimeras (the 2F5-presenting chimera, B1-16, and the 4E10-presenting chimeras, 02A1, 02B2, and 22C1) were able to elicit neutralizing antibodies against pseudoviruses of at least three subtypes). Interestingly, 12B1 compared poorly with the other chimeras, as measured by ELISAs and the MTT cell-killing assay. These measures of antigenicity, while not predictive of the best immunogens, nonetheless offer help in generally guiding our efforts toward the isolation and identification of the most valuable immunogens.

Given the evidence that a long CDR-H3 is essential for neutralization by 2F5 [79,80] and 4E10 [26], it is conceivable that 12B1 also elicited antibodies with long H3 loops and/or, possibly elicited polyreactive antibodies as well, improving its breadth in neutralizing HIV without improving its affinity to the W^670^NWFDITN^677^ sequence [26]. Another possibility is that the antibodies elicited were able to find an unusual angle of approach to the epitope and that the ability to access the epitope outweighed the importance of its affinity.

One advantage offered by the chimeric HRV system is that presentation of foreign epitopes on the VP2 puff of its neutralizing immunogenic site II allows for a number of possibilities that are less likely to occur in the context of native HIV. On one hand, with the freedom afforded by genetic engineering, one can generate epitope presentations that are more accessible. The value of this benefit was recently demonstrated by the identification of the new MPER mAb, 10E8 [81]. With its epitope consisting of N^671^WFDITNWLWYIR^683^, containing both the 4E10 and Z13e1 epitopes (though on different faces), this antibody binds with greater accessibility and affinity to the MPER than do mAbs 2F5, 4E10, and Z13e1, resulting in greater neutralizing potency against many HIV pseudoisolates. It could be that greater immunogenicity could have been achieved with the HRV: HIV gp41 MPER chimeras had some of them included the Y^681^, I^682^, and the critical R^683^. However, it is quite possible that the inclusion of the additional Y^681^IR^683^ could have been deleterious to the growth of chimeric viruses or caused misfolding of the epitope within the context of HRV. One could deduce that having an immunogen that presents crucial residues in an accessible way could lead to the production of antibodies that are predisposed to bind to somewhat less accessible residues when they do become more exposed. On another hand, tight homotypic bivalent binding across the two-fold axis of HRV [82] suggests that presentation of foreign epitopes that are not normally involved in tight bivalent binding by antibodies can now elicit such tightly binding antibodies, setting the stage for potential increases in antibody-binding affinity. While such high affinity bivalent binding to the MPER of HIV appears to be rare [83], genetic engineering (including the engineering of HRV chimeras) has the potential to expand the neutralization horizon (conceivably obviating the tendency for antibodies to make polyreactive contacts in such cases [84]). Given that the MPER itself is weakly immunogenic in native HIV-1 infection, improving neutralizing responses against this highly conserved region is a priority for the development of AIDS vaccines.

## Supporting Information

Table S1 **Propensities of linker amino acids for 4E10 Library III chimeras.** The residues expected correspond to the nucleotides encoded at the plasmid level. The residues observed are shown at both the N linker and C linker (beyond the N- and-K-biased residue adjacent to the 4E10 epitope). Residues for the groupings are as follows: α-helix promoters: E, L, A, V, and Y; β-sheet disrupters: N, P, G, and S; β-turn promoters: G, N, D, S, and P; β-turn or α-helix promoters: E, L, A, V, Y, G, N, D, S, and P.
(DOCX)
