# Correction: Chimeric Rhinoviruses Displaying MPER Epitopes Elicit Anti-HIV Neutralizing Responses

**DOI:** 10.1371/annotation/57fc0148-19fe-4b8b-b2f4-bb5f60baa971

**Published:** 2013-09-27

**Authors:** Guohua Yi, Mauro Lapelosa, Rachel Bradley, Thomas M. Mariano, Denise Elsasser Dietz, Scott Hughes, Terri Wrin, Chris Petropoulos, Emilio Gallicchio, Ronald M. Levy, Eddy Arnold, Gail Ferstandig Arnold

The captions of Fig 3 and Fig 4 were mixed up during the production period in the press. Therefore, they should be switched back to match the correct figures. 

