# Supplementary material for: Chimeric Rhinoviruses Displaying MPER Epitopes Elicit Anti-HIV Neutralizing Responses
Source: PLoS One. 2013 Sep 6;8(9):e72205. doi: 10.1371/journal.pone.0072205 (PMC3765159; doi:10.1371/journal.pone.0072205)
Supplement: Table S1 — Propensities of linker amino acids for 4E10 Library III chimeras. The residues expected correspond to the nucleotides encoded at the plasmid level. The residues observed are shown at both the N linker and C linker (beyond the N- and-K-biased residue adjacent to the 4E10 epitope). Residues for the groupings are as follows: α-helix promoters: E, L, A, V, and Y; β-sheet disrupters: N, P, G, and S; β-turn promoters: G, N, D, S, and P; β-turn or α-helix promoters: E, L, A, V, Y, G, N, D, S, and P. (DOCX) [file pone.0072205.s001.docx]

**Supporting Table**

**Table S1.** **Propensities of linker amino acids for 4E10 Library III chimeras.** The residues expected correspond to the nucleotides encoded at the plasmid level. The residues observed are shown at both the N linker and C linker (beyond the N- and-K-biased residue adjacent to the 4E10 epitope). Residues for the groupings are as follows: α-helix promoters: E, L, A, V, and Y; β-sheet disrupters: N, P, G, and S; β-turn promoters: G, N, D, S, and P; β-turn or α-helix promoters: E, L, A, V, Y, G, N, D, S, and P.

|  | **Plasmids** | **Selected chimeras** | | **Unselected chimeras** | |
| --- | --- | --- | --- | --- | --- |
|  | Expected | N linker observed | C linker observed | N linker observed | C linker observed |
| α-helix promoters | 53.1% | 30.0% | 70.6% | 38.7% | 60.5% |
| β-sheet disrupters | 25.0% | 30.0% | 70.6% | 29.0% | 52.6% |
| β-turn promoters | 26.6% | 70.0% | 40.0% | 48.4% | 31.6% |
| α-helix or β-turn promoters | 79.6% | 100% | 94.4% | 80.6% | 92.1% |
